# Expression and methylation patterns partition luminal-A breast tumors into distinct prognostic subgroups

**DOI:** 10.1186/s13058-016-0724-2

**Published:** 2016-07-07

**Authors:** Dvir Netanely, Ayelet Avraham, Adit Ben-Baruch, Ella Evron, Ron Shamir

**Affiliations:** Blavatnik School of Computer Science, Tel Aviv University, Tel Aviv, Israel; Oncology Department, Assaf Harofeh Medical Center, Tsrifin, Israel; Department of Cell Research and Immunology, George S. Wise Faculty of Life Sciences, Tel Aviv University, Tel Aviv, Israel

**Keywords:** Breast cancer subtypes, Luminal-A, Unsupervised analysis, Clustering, RNA-Seq, DNA methylation

## Abstract

**Background:**

Breast cancer is a heterogeneous disease comprising several biologically different types, exhibiting diverse responses to treatment. In the past years, gene expression profiling has led to definition of several “intrinsic subtypes” of breast cancer (basal-like, HER2-enriched, luminal-A, luminal-B and normal-like), and microarray based predictors such as PAM50 have been developed. Despite their advantage over traditional histopathological classification, precise identification of breast cancer subtypes, especially within the largest and highly variable luminal-A class, remains a challenge. In this study, we revisited the molecular classification of breast tumors using both expression and methylation data obtained from The Cancer Genome Atlas (TCGA).

**Methods:**

Unsupervised clustering was applied on 1148 and 679 breast cancer samples using RNA-Seq and DNA methylation data, respectively. Clusters were evaluated using clinical information and by comparison to PAM50 subtypes. Differentially expressed genes and differentially methylated CpGs were tested for enrichment using various annotation sets. Survival analysis was conducted on the identified clusters using the log-rank test and Cox proportional hazards model.

**Results:**

The clusters in both expression and methylation datasets had only moderate agreement with PAM50 calls, while our partitioning of the luminal samples had better five-year prognostic value than the luminal-A/luminal-B assignment as called by PAM50. Our analysis partitioned the expression profiles of the luminal-A samples into two biologically distinct subgroups exhibiting differential expression of immune-related genes, with one subgroup carrying significantly higher risk for five-year recurrence. Analysis of the luminal-A samples using methylation data identified a cluster of patients with poorer survival, characterized by distinct hyper-methylation of developmental genes. Cox multivariate survival analysis confirmed the prognostic significance of the two partitions after adjustment for commonly used factors such as age and pathological stage.

**Conclusions:**

Modern genomic datasets reveal large heterogeneity among luminal breast tumors. Our analysis of these data provides two prognostic gene sets that dissect and explain tumor variability within the luminal-A subgroup, thus, contributing to the advancement of subtype-specific diagnosis and treatment.

**Electronic supplementary material:**

The online version of this article (doi:10.1186/s13058-016-0724-2) contains supplementary material, which is available to authorized users.

## Background

Breast cancer is a heterogeneous disease exhibiting high tumor variability in terms of the underlying biological mechanisms, response to treatment, and overall survival rate [1]. Accurate identification of the unique biological features characterizing each subtype is pivotal for improving our understanding of the disease, identifying subtype-specific biomarkers, targeted drug development, and better prediction of response to treatment.

Originally, therapeutic decisions in breast cancer were guided by clinicopathologic parameters like tumor size, presence of lymph-node/remote metastases, and histological grade. In addition, the status of three immunohistochemistry biomarkers - estrogen receptor (ER), progesterone receptor (PR), and human epidermal growth factor receptor 2 (*HER2/ERBB2*) allowed the development of targeted therapies and proved predictive of treatment response [2].

With the emergence of global molecular profiling techniques, large genomic datasets became available for subtype discovery using unsupervised algorithms. By this methodology, breast samples are partitioned into subgroups using clustering algorithms, such as hierarchical clustering [3] or K-Means, and then subgroup significance is evaluated using the clinical data associated with the samples.

Initially, microarray data were used to define four molecular breast cancer subtypes (basal-like, HER2-enriched, luminal and normal-like) based on characteristic gene expression signatures in correlation with clinical data [4]. These molecular subtypes correlated reasonably well with the immunohistochemical biomarker-based classification. Thus, basal-like samples are mostly triple-negative (ER-/PR-/Her2-), luminal samples are mostly ER+, and HER2-enriched tumors are characterized by amplification and high expression of the *HER2/ERBB2* gene [5, 6].

Subsequent analysis conducted on a larger dataset separated the luminal subtype into two distinct subgroups named luminal-A and luminal-B. Luminal-B tumors have higher expression of proliferation genes including Ki-67, and confer worse prognosis [7–9]. Moreover, luminal-B tumors respond better to chemotherapy, while patients with luminal-A cancer gain most benefit from antiestrogen treatment [10].

As the partitioning of breast tumors into five molecular subtypes has gained acceptance and popularity, several expression-based predictors have been developed. A central predictor is PAM50, which maps a tumor sample to one of the five subtypes based on the gene expression pattern of 50 genes [11]. Though expected to be more robust than traditional classification systems that rely only on a few biomarkers, the separation between luminal-A and luminal-B by the various predictors is not consistent, suggesting that these molecular subtypes may not represent distinct coherent sample groups [12].

Other attempts to classify breast tumors were based on other profiling technologies such as miRNA arrays [13, 14], copy number variations [15] or a combination of several different technologies [16, 17]. The various studies have different levels of agreement with the expression-based molecular subtypes, but taken together they strongly indicate the existence of additional, subtler subtypes than the PAM50 subtypes [18].

Epigenetic modifications such as DNA methylation arrays, which measure the methylation status of thousands of CpG sites across the genome [19], were also used for breast cancer classification. DNA methylation changes were shown to play a pivotal role in cancer initiation and progression [20, 21]. Particularly, promoter hyper-methylation was associated with silencing of tumor suppressor genes [22]. Several studies associated breast cancer molecular subtypes with specific methylation patterns [23], while others showed that methylation data may reveal additional complexity not captured at the expression level, possibly identifying finer patient groups of clinical importance [24].

The large breast cancer dataset developed and provided by The Cancer Genome Atlas project [25] includes more than a thousand breast tumor samples characterized by various modern high-throughput genomic technologies. This dataset constitutes a significant leap forward compared to the older microarray-based data. mRNA abundance levels are measured in TCGA dataset using the RNA-Seq technology. This technology has increased sensitivity and a higher dynamic range compared to microarrays [20, 21]. DNA-methylation arrays applied on the same samples can help decipher biological tumor variability by epigenetic modifications not manifested at the gene expression level.

The aim of this study was to improve the classification of breast tumors based on the extensive TCGA expression and methylation data that have recently become available. We utilized these datasets to revisit the current classification of breast tumors into biologically distinct subgroups. Our improved and refined classification may contribute to the precision of diagnosis and thus, to more personalized treatment.

## Methods

### Study objectives

Our initial question was whether unsupervised clustering of all TCGA breast samples using the RNA-Seq data would reconstruct the partition defined by PAM50. As the luminal samples had the highest variability in our global clustering, we also asked how the luminal samples would cluster into two groups based on the RNA-Seq data, how the resulting sample groups would compare to the PAM50 partition into luminal-A and luminal-B, and whether that partition would have a clinical advantage over the PAM50 partition of the luminal samples. Looking into the internal structure of the highly variable luminal-A samples, we asked whether this PAM50 group can be further partitioned into finer subgroups with biological distinctness and clinical significance. We then used enrichment analysis to explore the biological mechanisms underlying the new luminal-A subgroups.

We asked similar questions about breast tumor variability at the epigenetic level. We evaluated the methylation-based partition of all breast tumors, all the luminal samples and the highly heterogeneous luminal-A, and compared the resulting partitions to PAM50. To examine the biological characteristics of differentially methylated CpGs (DMCs) separating the new methylation-based luminal-A subgroups, we conducted enrichment analysis. Finally, we performed multivariate COX survival analysis to determine whether the new subgroups have independent prognostic value.

### Data acquisition and preprocessing

TCGA data on invasive carcinoma of the breast were downloaded from the UCSC Cancer Browser web site [26] together with accompanying clinical information. The downloaded RNA-Seq gene expression dataset (Illumina HiSeq platform, gene level RSEM-normalized [27], log2 transformed) included 1215 samples of which 11 samples from male patients, 8 metastatic samples, and 30 samples of unknown tissue source were filtered out. PAM50 calls (obtained directly from UNC, including PAM50 proliferation scores) were available for 1148 of the filtered samples, and were distributed as follows: 183 basal-like, 78 HER2-enriched, 534 luminal-A, 203 luminal-B and 150 normal-like.

We also downloaded DNA methylation profiles (Illumina Infinium Human Methylation 450K platform, beta values) [19] containing 872 samples of which 8 male samples, 5 metastatic samples and 19 samples of unknown tissue source were filtered out. We used only 679 tumor samples for which PAM50 calls were available, including 124 basal-like, 42 HER2-enriched, 378 luminal-A and 135 luminal-B samples. Our analysis used only the 107,639 probes of the Infinium-I design type for which a gene symbol was available. This allowed us to bypass the bias of the two probe designs included on the array, to focus on differentially methylated sites that are associated with known genes, and also to reduce the number of analyzed features.

### Unsupervised analysis of the tumor samples

Unsupervised analysis of the various sample subsets was executed by clustering the samples based on the 2000 features (genes or CpGs) showing the highest variability over the samples included in each analysis. We used the K-Means clustering algorithm in Matlab (release 2015a) with correlation distance and 100 replicates from which a solution minimizing the sum of point-to-centroid distances was chosen. Due to the high variability among sample subgroups in the breast cancer datasets, reselecting the top variable genes for the analysis of each sample set (and renormalizing accordingly) is crucial to ensure use of the features most relevant to that set. Each feature was independently centered and normalized over the analyzed samples prior to clustering.

Cohort descriptions for the samples used in each analysis are provided in Additional file 1 (Tables S-1A, S-2A, S-3A for the RNA-Seq analyses and Tables S-6A, S-7A and S-8A for the DNA methylation analysis). The TCGA sample Ids included in each analysis are listed in Additional file 2.

### Sample cluster enrichment and survival analysis

To evaluate the clinical relevance of the sample clusters obtained in each unsupervised analysis, we used the extensive clinical information available from TCGA for each sample. Enrichment significance of sample clusters for categorical variables (such as the PAM50 subtype or histological type) was calculated using the false discovery rate (FDR)-corrected hypergeometric test. For numeric variables (such as age, percent tumor nuclei, and others) the difference between sample groups was evaluated using the Wilcoxon rank–sum test (Mann–Whitney *U* test).

Survival and recurrence-free survival curves were plotted using the Kaplan-Meier estimator [28] and *p* values for the difference in survival for each group versus all other groups were calculated using the log-rank (Mantel–Haenszel) test [29, 30]. Cox univariate and multivariate survival analyses were conducted using Matlab implementation; *p* values were corrected using FDR. The analysis and visualization scripts are publicly available as an interactive graphical tool named PROMO [31].

### Analysis of differentially expressed genes and gene enrichment

A list of genes that have the highest differential expression between the two RNA-Seq-based sample groups LumA-R1 and LumA-R2 was generated by applying the Wilcoxon rank–sum test on all dataset genes exhibiting non-zero variance (n = 19,913) after flooring all dataset values to 1 and ceiling to 14. We selected the 1000 genes exhibiting the most significant *p* values that also have a median difference of at least 0.5 (log2-transformed RSEM expression values). All genes on the list had significantly higher expression in the LumA-R2 sample group (the lowest *p* value was 8.1e-28).

Gene enrichment tests were performed on these 1000 genes against a background of all genes included in the rank–sum test. The Expander software suite [32, 33] was used to detect significant enrichments for Gene Ontology (GO) [34], Kyoto Encyclopedia of Genes and Genomes (KEGG) pathways [35], Wiki-Pathways [36] and chromosomal location enrichments. GO tests were also performed using the GOrilla tool [37]. The list of 1000 top differentially expressed genes and detailed results of the enrichment analysis are provided in Additional file 3.

### Analysis of differentially methylated CpGs, correlation to expression and CpG enrichment

To identify CpGs that are differentially methylated between LumA-M1 and LumA-M3 samples we applied the rank–sum test on all CpGs that survived our preprocessing and also had non-zero variability in the relevant samples (n = 93,880). We then selected the 1000 CpGs that had the highest significance and a minimal median difference of 0.2 (in Beta values). All selected CpGs had significantly higher mean methylation in the LumA-M1 compared to the LumA-M3 group.

To focus on DMCs with genes that had concomitant changes in expression, we calculated Spearman correlation between each CpG and the expression profile of its associated gene based on the Illumina probe-set annotation. The correlation values enabled the identification of 586 DMCs (rank–sum *p* value <0.01, median difference >0.2) negatively correlated to expression (*R* < -0.2) and a second smaller group of 212 DMCs positively correlated (*R* > 0.2) with expression.

We used the array CpG annotations provided by Illumina to calculate enrichment of each one of the three CpG lists (top 1000 DMCs, 586 negatively correlated DMCs and 212 positively correlated DMCs) for features like differentially methylated regions (DMRs), enhancer regions, UCSC RefGene groups and regulatory feature groups. Gene enrichment analysis was performed on the unique genes composing each CpG list, using the Expander and Gorilla tools as described above. Enrichment for InterPro [38] terms was calculated using the Database for Annotation, Visualization and Integrated Discovery (DAVID) [39]. Enrichment for tumor suppressor genes was calculated by hypergeometric test based on the TSGene [40] catalog. The lists of differentially methylated CpGs in addition to detailed results of the enrichment analysis are provided in Additional files 4, 5 and 6.

## Results

### Separation of luminal-A and luminal-B samples is not reconstructed by RNA-Seq unsupervised analysis

**Fig. 1:**
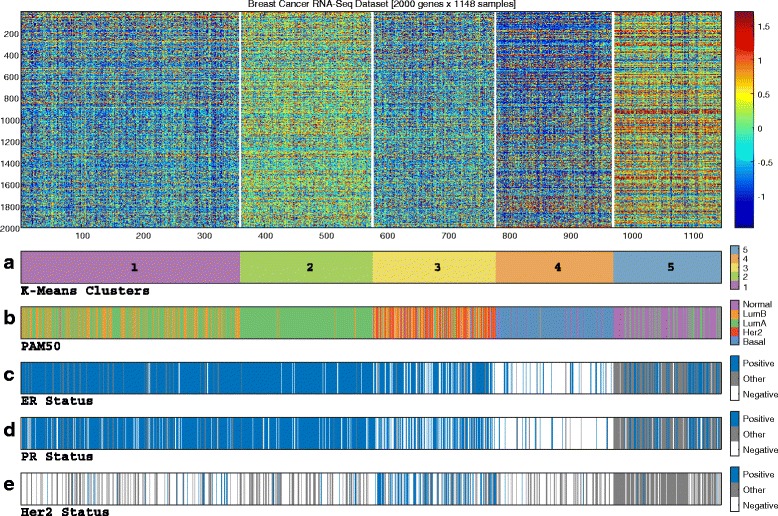
Global unsupervised clustering of 1148 breast samples using RNA-Seq data. Applying the K-Means algorithm using K = 5 on the RNA-Seq dataset yielded a partition exhibiting moderate agreement with PAM50 labels and the three immunohistochemical markers. Notably, luminal-A samples were split between a rather homogenous cluster 2 and cluster 1, which is composed of a mix of luminal-A and luminal-B. **a** K-Means clusters. **b** PAM50 calls. **c** Estrogen receptor (*ER*) status. **d** Progesterone receptor (*PR*) status. **e** Human epidermal growth factor receptor 2 (*HER2*) status

We started by evaluating the global sample structure within the RNA-Seq gene expression data obtained from TCGA. We applied unsupervised analysis on both tumor (n = 1035) and normal (n = 113) breast samples using the K-Means clustering algorithm over the top 2000 variable genes. As our initial goal was to compare the resulting partition into the four intrinsic molecular types, we used K = 5 (corresponding to the four types represented by PAM50 label classes in addition to normal). The results are shown in Fig. 1.

The resulting clusters exhibited moderate correspondence with PAM50 labels: most basal-like, normal and HER2-enriched samples fell into three different clusters (numbers 4, 5, and 3, respectively, listed in decreasing levels of homogeneity), whereas the luminal samples exhibited much greater variability. Importantly, most luminal-A sample were split between two different clusters - a homogenous luminal-A cluster (cluster 2), and a cluster composed of a mix of luminal-A and luminal-B samples (cluster 1).

Furthermore, the samples assigned to cluster 2 exhibited a very distinct expression pattern, overexpressing 1184 genes compared to cluster 1 (out of the 1421 differentially expressed genes, see “Methods”). Cluster 1 samples overexpressed only 229 genes compared to cluster 2 (see Additional file 1: Figure S-1E for per-cluster distribution and Additional file 1: Figure S-1F for results of differential gene expression analysis).

According to these results, the variability within the luminal samples is not sufficiently captured by the PAM50 luminal-A and luminal-B subtypes. Specifically, they suggest that luminal-A samples can be further partitioned into finer subgroups, possibly having clinical meaning.

### Unsupervised partition of luminal samples predicts survival and recurrence better than PAM50

**Fig. 2:**
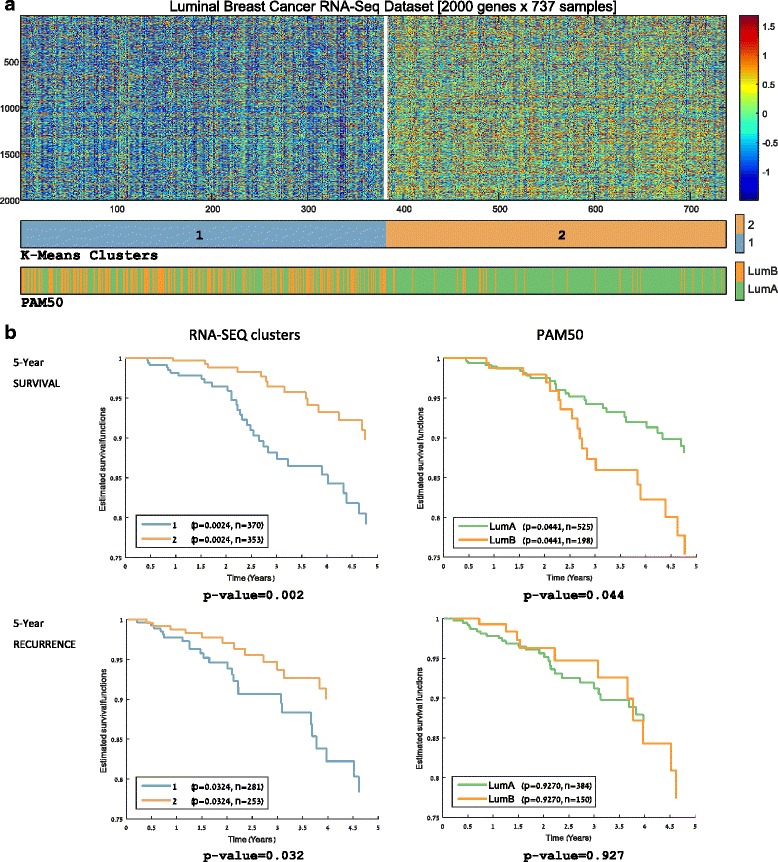
Unsupervised analysis of luminal breast samples using RNA-Seq data. **a** Applying the K-Means algorithm on the 737 luminal samples using K = 2 splits the samples into two subgroups exhibiting better five-year prognostic value than the PAM50 luminal-A/luminal-B partition. **b** Five-year survival and recurrence for the two luminal breast cancer partitions. The partition into two RNA-Seq-based clusters outperforms PAM50 partition of the luminal samples in both survival and recurrence. *P* values were calculated using the log-rank test

To further investigate the variability among luminal samples, we clustered the 737 luminal samples (534 luminal-A and 203 luminal-B samples based on PAM50 labels) into two groups. The results are shown in Fig. 2a. Similar to the global analysis, the luminal-A samples were divided between a luminal-A mostly homogenous cluster (cluster 2) and a cluster composed of both luminal-A and luminal-B samples (cluster 1).

Survival analysis performed on the two luminal partitions (the PAM50 luminal-A/luminal-B partition, and the two K-Means clusters shown in Fig. 2a) showed that the RNA-Seq-based clustering partition outperforms the luminal-A/luminal-B distinction in terms of both survival and recurrence (5-year survival plots are shown in Fig. 2b; also see Additional file 1: Figure S-2A for overall survival plots). Hence, the signal identified by our unsupervised analysis of the RNA-Seq data translates into a clinically relevant partition of the luminal samples that has better predictive power than the PAM50 luminal-A/luminal-B partition in terms of both survival and recurrence.

### Luminal-A samples have two distinct classes exhibiting clinical significance

**Fig. 3:**
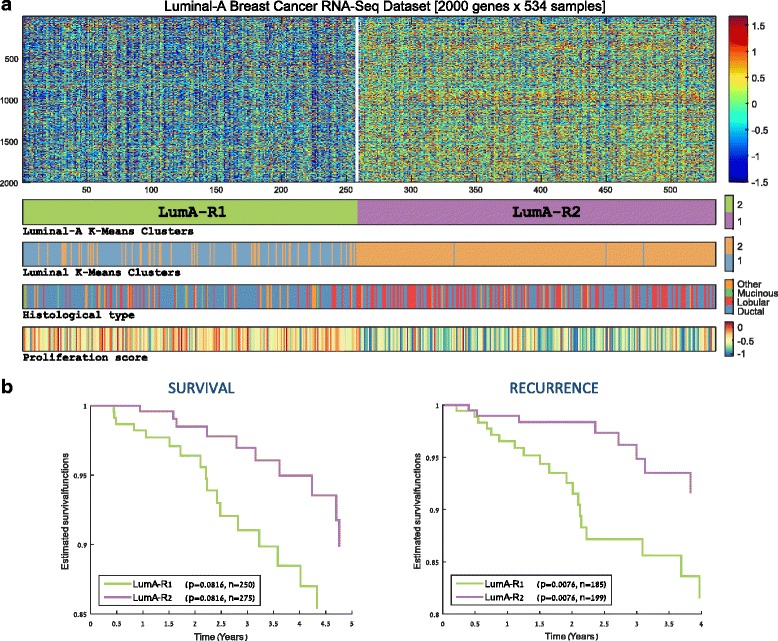
Unsupervised analysis of luminal-A (*LumA*) breast samples. **a** Clustering of 534 RNA-Seq profiles partitions the data into two groups exhibiting distinct expression profiles. The clusters also show significant enrichment for clinical variables including recurrence, proliferation score, age, and histology. The *bars* below the heatmap show, from *top* to *bottom*, the partition of the samples, the designation of the samples according to the clustering of all luminal samples (see Fig. 2), histological type, and proliferation scores. **b** Five-year survival and recurrence analysis in the two luminal-A subgroups. LumA-R2 samples exhibit significantly reduced five-year recurrence rate compared with LumA-R1

As the luminal-A samples displayed the highest level of variability by consistently falling into two major subgroups in previous steps, we focused on this PAM50 class in an attempt to explore its underlying substructures. To this end, we re-clustered only the 534 luminal-A samples into two groups (Fig. 3a). As the resulting clusters were found to be significantly enriched for various clinical variables, we designated them as LumA-R1 (n = 258) and LumA-R2 (n = 276).

The most apparent property of the resulting partition was the general overexpression pattern in LumA-R2 samples compared to LumA-R1 samples. Indeed, out of the 2000 genes selected for clustering, 1276 were differentially expressed and 1068 of them were overexpressed in LumA-R2 samples (based on the FDR-corrected rank-sum test). A very similar partition (chi-square, *p* = 1.1e-40) with a parallel overexpression pattern was identified on a microarray gene expression dataset also available from TCGA for a subset of the luminal-A samples used here (n = 265). This supports the conclusion that the partition and distinct overexpression pattern we observed are not an artifact originating from RNA-Seq measurement technology or from any normalization protocols applied on the dataset (see Additional file 1, section 4).

Recurrence analysis performed on these two luminal-A subgroups identified that LumA-R2 samples were associated with a significantly reduced 5-year recurrence rate (*p* = 0.0076, Fig. 3b). Enrichment analyses on additional clinical information available for the samples revealed that LumA-R1 and LumA-R2 subgroups are enriched with ductal (*p* = 2.1e-05) and lobular (*p* = 9.7e-12) histological types, respectively. LumA-R1 samples were associated with a higher proliferation score (*p* = 8.9e-25), older age (*p* = 2.6e-05), and a slight but significant decrease in normal cell percent (*p* = 2.8e-08) accompanied by an increase in tumor nuclei percent (*p* = 2.6e-12) compared with LumA-R2 samples (see Table 1).

**Table 1:** The main characteristics distinguishing between the luminal-A subgroups, LumA-R1 and LumA-R2

Group characteristic	LumA-R1	LumA-R2	*P* value
Recurrence-free survival	Increased recurrence	Reduced recurrence	7.6e-3
Histological type	Ductal (*p* = 2.1e-05)	Lobular (*p* = 9.7e-12)	
Age, years, average	61.5	57.4	2.6e-05
Proliferation score	-0.4	-0.6	8.9e-25
Tumor nuclei percent	80 %	73 %	2.6e-12
Normal cell percent	2.9 %	6.1 %	2.8e-08
Gene overexpression	194	1068	

Average values are shown for each group where relevant. Gene overexpression is computed with respect to the 2000 genes used for clustering.

Comparing the luminal-A partition shown in Fig. 3a to the groups formed when clustering all the luminal samples (Fig. 2a), we note that almost all LumA-R2 samples are contained within cluster 2 (composed of mainly luminal-A samples), whereas most LumA-R1 are contained within cluster 1 (composed of a mix of luminal-A and luminal-B samples) (see the second label bar in Fig. 3a). This suggests that LumA-R1 samples are more similar in their expression profile to luminal-B samples compared with LumA-R2 samples.

### Luminal-A subgroups exhibit distinct immune system expression profiles

In order to identify genes that distinguish best between LumA-R1 and LumA-R2 samples, we created a list of the 1000 most differentially expressed genes (see “Methods”). In agreement with the general expression pattern described earlier, all genes in the list were overexpressed in LumA-R2 compared to LumA-R1 samples. The most significant categories in the enrichment analysis performed in this list were related to the immune system regulation. The more specific category of T cell receptor signaling genes appeared consistently in analyses based on various annotation databases (Gene Ontology: "T Cell activation" *p* = 1e-05, KEGG Pathway: "T Cell receptor signaling pathway" *p* = 3e-07, Wiki-Pathway: "T Cell receptor (TCR) Signaling Pathway" *p* = 1.09e-07). Other enrichments of interest included the KEGG Pathways "Cytokine-cytokine receptor interaction" (p = 2.13e-13), "Chemokine signaling pathway" (*p* = 1.14e-09) and Wiki-Pathway "B Cell Receptor Signaling Pathway" (*p* = 1.72e-06). See Table 2 for a list of the most significant categories, and Additional file 1, section 5 for the full list.

**Table 2:** The most enriched functional categories among the 1000 genes most differentially expressed between LumA-R1 and LumA-R2 samples

Enrichment type	Term	Number of genes	*P* value
Gene Ontology	Regulation of immune system process	152	3.74e-50
	Immune system process	201	3.65e-47
	Regulation of leukocyte activation	71	2.37e-28
	Regulation of multicellular organismal process	183	2.89e-28
	Cell activation	91	4.59e-28
	Regulation of response to external	73	8.18e-27
	Regulation of biological quality	218	1.82e-26
	Leukocyte activation	67	1.95e-26
	Positive regulation of cell activation	56	5.13e-24
	T cell activation	45	4.93e-22
	Regulation of cell proliferation	128	1.83e-21
KEGG Pathways	Cytokine-cytokine receptor interaction	56	4.76e-22
	Hematopoietic cell lineage	29	1.50e-17
	Cell adhesion molecules (CAMs)	30	4.08e-13
	Primary immunodeficiency	16	8.70e-13
	Chemokine signaling pathway	31	1.14e-09
	Complement and coagulation cascades	17	1.36e-08
	T cell receptor signaling pathway	20	1.30e-07
	Allograft rejection	11	6.44e-07
	Natural killer cell mediated cytotoxicity	20	5.66e-06
	Pathways in cancer	34	1.49e-05
Wiki-Pathways	TCR signaling pathway	10	1.55e-09
	B cell receptor signaling pathway	10	1.72e-06
	Focal adhesion	11	5.88e-05
	Complement activation, classical pathway	6	8.38e-05
Chromosomal location	11q23	18	1.84e-05
	Xq23	8	4.99e-05

All the genes on the list showed significantly higher expression on the LumA-R2 samples compared to LumA-R1 samples

**Fig. 4:**
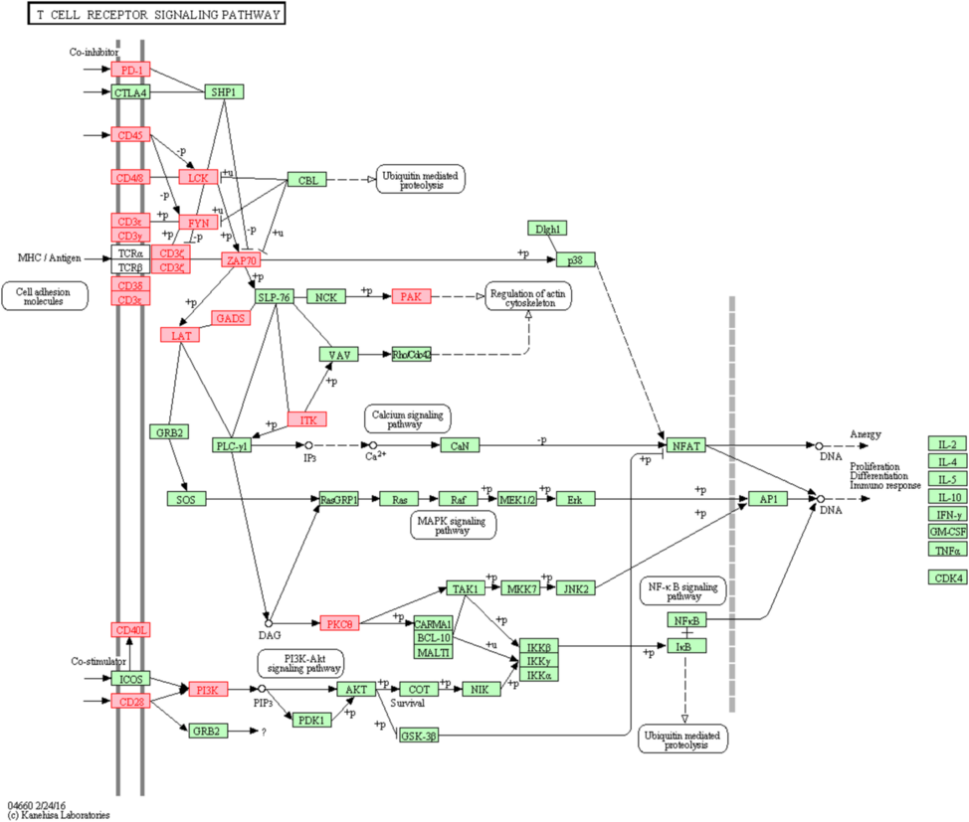
LumA-R2 samples overexpress genes in the T cell receptor signaling pathway. The list of top 1000 genes differentially expressed in LumA-R1 and LumA-R2 samples was found to be significantly enriched for the pathway genes (*p* = 1.3e-07). Genes marked in *red* are overexpressed in LumA-R2 samples. Pathway and graphics were taken from the Kyoto Encyclopedia of Genes and Genomes (KEGG) database

Careful examination of the gene list revealed that LumA-R2 samples overexpress genes that are typically expressed by various immune system cells (e.g., the leukocyte marker *CD45/PTPRC*, T cell marker *CD3*, and B cell marker *CD19*) [41–44]. A significant number of overexpressed genes are related to the T cell receptor (*CD3D*, *CD3E*, *CD3G*, and *CD247*) and the upstream part of its signaling pathway (ZAP70, LCK, FYN, LAT, PAK, and ITK) [45] (Fig. 4). Interestingly, the overexpressed genes were related to T cell or natural killer (NK)-mediated cytotoxic activities (*GZMA*, *GZMB*, *GZMH*, *GZMM*, and *PRF1*) [46, 47].

We also observed that the overexpression of immune receptor genes in LumA-R2 samples was accompanied by overexpression of several chemokine genes (*CCL5*, *CCL17*, *CCL19*, and *CCL21*) and their corresponding receptors (*CCR5*, *CCR4*, and *CCR7*). Topping the list of overexpressed genes in Lum-A-R2 samples (ranked by *p* value) is the Interleukin-33 (*IL-33*) gene, which drives T helper 2 (Th2) responses [48]. In summary, LumA-R2 samples exhibit better prognosis based on several clinical parameters while overexpressing a significant number of genes related to the immune system.

### Analysis of DNA methylation identifies a luminal subgroup characterized by hyper-methylation and a significantly poorer outcome

The luminal-A tumors proved to be the most heterogeneous in our gene expression analysis. To further identify and characterize clinically meaningful subgroups within the luminal-A group, we explored breast tumor variability on the epigenetic level as well.

**Fig. 5:**
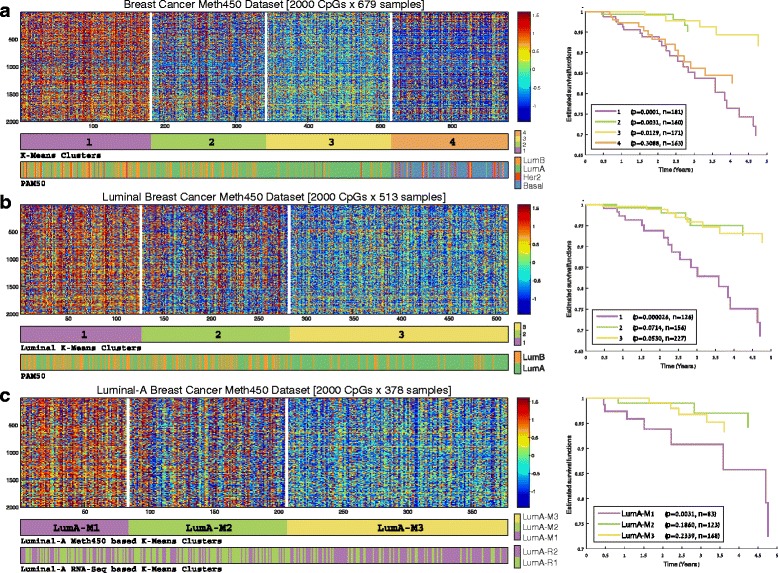
Unsupervised analysis of breast cancer tumors using DNA methylation data. Samples were clustered by K-Means based on correlation using the top 2000 variable CpGs over each sample subset. **a** All 679 tumors. **b** The 579 samples identified as luminal-A and luminal-B by PAM50 classification. **c** The 378 luminal A samples only. *First bar* below each expression matrix shows the assignment of the samples to methylation-based clusters. *Second bar* (**a** and **b**) shows PAM50 calls for the samples. *Second bar* (**c**) presents the RNA-Seq based LumA-R1/2 subgroups defined in Figure 3. *Right panels* show five-year Kaplan-Meier survival plots for the resulting groups

Using the Methylation 450K array dataset available from TCGA, we started our analysis as in the expression data, by clustering all 679 tumor samples into four groups, corresponding to the number of PAM50 classes. The resulting clusters (Fig. 5a) had modest agreement with the expression-based PAM50 classes; all basal-like samples were assigned to a single cluster exhibiting a distinct hypo-methylation pattern (cluster 4), whereas HER2-enriched samples were scattered over three different clusters, indicating that this subtype has reduced manifestation at the methylation level. Notably, most luminal samples were assigned to three different clusters (1–3) with methylation-level gradation on the top 2000 variable CpGs. Cluster 1 exhibited a strong hyper-methylation pattern, contained the highest ratio of luminal-B samples, and was associated with significantly poorer survival compared to the three other clusters (*p* = 0.0001). Cluster 3, on the other hand, exhibited opposite characteristics: lower methylation levels, the lowest ratio of luminal-B samples and a better outcome (*p* = 0.0129).

Similar results were obtained when we clustered only the 513 luminal A and B samples (Fig. 5b). Here we used the top 2000 variable genes within these samples, to remove the effect of the other two subtypes on the clustering. Importantly, out of the 127 samples comprising the hyper-methylated cluster 1, which was associated with reduced survival (*p* = 2.6e-05), 76 samples were labeled as luminal-A, a subtype usually associated with good survival. In other words, approximately 20 % of the 378 luminal-A samples (as called by the expression-based PAM50) included in the analysis, could actually be assigned to a higher risk group based on methylation data (see Additional file 1, section 7 for more details).

The three-way partition by methylation levels and its association to differential survival risk also appeared when we repeated the analysis in the group of 378 luminal-A samples, using the top 2000 variable CpGs on these samples (Fig. 5c). The three methylation-based luminal-A clusters were designated LumA-M1, LumA-M2 and LumA-M3. The 84-sample LumA-M1 cluster (comprising approximately 22 % of the luminal-A samples) was associated with significantly reduced 5-year survival (*p* = 0.0031).

Furthermore, the methylation-based partitioning of the luminal-A samples (LumA-M1/2/3) correlated significantly with the expression-based partitioning (LumA-R1/2, chi-square *p* = 4.4e-08). The LumA-M2 cluster was enriched for LumA-R1 samples (*p* = 1.4e-06) and the LumA-M3 cluster was enriched for LumA-R2 samples (*p* = 1.6e-08), showing that the expression and the methylation-based patterns are related (see lower bar on Fig. 5c). Overall, we identified a poorer outcome subgroup within the luminal-A subtype, which is distinguished by a robust hyper-methylation pattern.

### Analysis of differentially methylated CpGs between the LumA-M1 and LumA-M3 subgroups and their correlation to gene expression

To uncover the biological features characterizing the distinct methylation patterns observed in the luminal-A subgroups, we examined the 1000 top DMCs (see “Methods”) between the hyper-methylated LumA-M1 (n = 84) and the hypo-methylated LumA-M3 (n = 171). These two sample subgroups represent the two extremes of the methylation gradient observed in the luminal-A samples. Of note, all 1000 top DMCs (representing 483 genes) were hyper-methylated in the LumA-M1 samples compared to LumA-M3 samples.

Gene enrichment analysis associated these 483 genes hyper-methylated on LumA-M1 samples with GO terms related to development, signaling, cell differentiation and transcription regulation (*p* < 1e-15). The genes were also enriched for the homeobox InterPro term (*p* = 3.6e-35), in line with previous reports describing the methylation of homeobox genes during breast tumorigenesis [49–51]. Further, the 483 genes were enriched for tumor suppressor genes according to the TSGene catalog [40] (*p* = 1.5e-03), including 48 such genes (see column 1 in Table 3). Analysis for CpG features of the top 1000 DMCs revealed significant enrichment for enhancer elements, tissue-specific promoters and cancer-specific DMRs (see column 1 in Table 4).

**Table 3:** Gene enrichment in the three subsets of CpGs exhibiting differential methylation between the LumA-M1 and LumA-M3 subgroups

	(1)		(2)		(3)	
	Hyper-methylated CpGs		Negative: *R* < -0.2		Positive: *R* > 0.2	
	1000 CpGs, 483 genes		586 CpGs, 340 genes		212 CpGs, 125 genes	
	Term	*P* value	Term	*P* value	Term	*P* value
Gene Ontology	Anatomical structure development	6.1e-28	Developmental process	7.8e-06	Pattern specification process	1.1e-13
	Developmental process	2.0e-25	Single organism signaling	2.4e-05	Regionalization	1.1e-12
	Multicellular organismal process	9.6e-24	Signaling	1.8e-05	Anatomical structure development	2.2e-11
	Single multicellular organism process	1.6e-22	Cellular developmental process	1.4e-05	Single organism developmental process	1.9e-11
	Single organism signaling	1.7e-21	Single organism developmental process	2.3e-05	Anatomical structure morphogenesis	1.8e-11
	Signaling	1.9e-21	Anatomical structure development	8.0e-05	Developmental process	1.7e-11
	Cell-cell signaling	1.7e-21	Cell-cell signaling	1.8e-04	Embryonic morphogenesis	1.1e-10
	Neuron differentiation	1.2e-20	Cell differentiation	2.2e-04	Cellular developmental process	1.8e-10
	Single organism developmental process	1.4e-19	Synaptic transmission	4.4e-04	Organ development	5.3e-10
	Regulation of transcription from RNA polymerase II promoter	1.2e-16	Anatomical structure morphogenesis	6.1e-04	Single multicellular organism process	5.6e-10
InterPro	Homeobox	3.6e-35	Homeobox	1.1e-04	Homeobox	2.1e-31
Tumor suppressor genes (TSGene 2.0)	AHRR, AKR1B1, BMP2, C2orf40, CDH4, CDO1, CDX2, CNTNAP2, CSMD1, DLK1, DSC3, EBF3, EDNRB, FAT4, FOXA2, FOXC1, GALR1, GREM1, GRIN2A, ID4, IRF4, IRX1, LHX4, MAL, MIR124-2, MIR124-3, MIR125B1, MIR129-2, MIR137, MIR9-3, ONECUT1, OPCML, PAX5, PAX6, PCDH8, PHOX2A, PRKCB, PROX1, PTGDR, RASL10B, SFRP1, SFRP2, SHISA3, SLIT2, SOX7, TBX5, UNC5D, ZIC1	1.5e-03	AKR1B1, ASCL1, BIN1, BMP4, CCDC67, CDK6, CDO1, EBF3, GSTP1, ID4, IRX1, L3MBTL4, LRRC4, MAP4K1, MME, NTRK3, PCDH10, PDLIM4, PROX1, PTGDR, RUNX3, SCGB3A1, SFRP1, SLC5A8, SLIT2, UBE2QL1, UNC5B, VIM, WT1	9.7e-02	AMH, GATA4, HOPX, HOXB13, LHX4, LHX6, MAP4K1, ONECUT1, PAX5, RASAL1, TBX5, TP73, WT1, ZIC1	5.5e-02
		(48 genes)		(29 genes)		(14 genes)

**Table 4:**
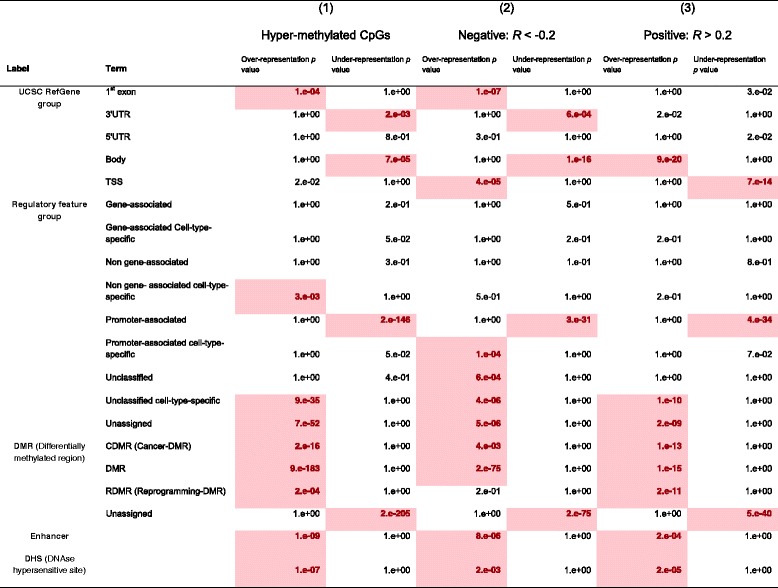
Feature enrichment in the three subsets of differentially methylated CpGs in LumA-M1 and LumA-M3 subgroups

CpG enrichment tests show that hyper-methylated CpGs negatively correlated with gene expression are enriched for upstream gene parts, whereas positively correlated CpGs are enriched for the gene body. All three hyper-methylated CpG groups are enriched for informatically determined enhancer elements and experimentally determined differentially methylated regions and DNAse hypersensitive sites. The *p* values represent hyper-geometric-based over-representation or under-representation and are FDR corrected (significant *p* values are marked in bold). UTR untranslated region, DMR differentially methylated region

The databases Gene Ontology, InterPro and Tumor Suppressor Genes 2.0 were used to test the hyper-methylated genes for enrichment. Group 1 is composed of the 1000 top differentially methylated CpGs with a mean difference of at least 0.2. All the CpGs on this list had significant hyper-methylation in the LumA-M1 samples compared to LumA-M3 samples. Group 2 is composed of the 586 CpGs with a differential methylation *p* value <0.01, a methylation mean difference >0.2 and Spearman-based correlation with expression <0.2. Group 3 is composed of 212 CpGs with a differential methylation *p* value <0.01, a methylation mean difference >0.2 and Spearman-based correlation with expression >0.2.

As DNA-methylation is known to regulate gene expression and as hyper-methylation of promoters is associated with gene silencing in cancer [52], we focused on LumA-M1 hyper-methylated CpGs that affect the expression of their corresponding genes. To this end, we used the RNA-Seq-based expression data available from TCGA for the same 378 analyzed samples to generate a second list of CpGs that are both hyper-methylated in LumA-M1 samples (differential methylation *p* < 0.01, median difference of 0.2) and that have methylation levels inversely correlated to the expression level of their corresponding gene (Spearman correlation *R* < -0.2). As can be seen in Table 4, the 586 CpGs that passed this filter (corresponding to 340 genes) had significant over-representation of upstream parts of their corresponding genes (UCSC RefGene Group: TSS and 1^st^ exon *p* < =4.4e-05) and under-representation of gene body (*p* = 1.43e-16) and 3'UTR (*p* = 5.83e-04). In terms of the regulatory feature group, these 586 CpGs had over-representation of "Promoter Associated Cell type specific" elements (*p* = 1.40e-04) accompanied by highly significant under-representation of "Promoter Associated" elements (*p* = 2.94e-31), suggesting that the observed hyper-methylation pattern involves tissue-specific promoters. Among the 340 under-expressed genes containing the 586 hyper-methylated CpGs, there were several tumor suppressor genes with under-expression that has previously been observed in breast cancer, such as *L3MBTL4* [53], *ID4* [54], *RUNX3* [55, 56], *PROX1* [57], *SFRP1* [58] and others. Gene-level and CpG-level enrichment for the negative correlations are shown in column 2 of Tables 3 and 4, respectively.

Interestingly, the 212 LumA-M1 hyper-methylated CpGs that were positively correlated with expression (Spearman *R* > 0.2) had higher enrichment of development-related GO terms compared with negatively correlated CpGs ("pattern specification process" *p* = 1.07e-13, "embryonic morphogenesis" *p* = 1.05e-10, "cell fate commitment" *p* = 5.49e-10). In contrast to the negatively correlated CpGs, they had high over-representation of "gene body" and under-representation of "TSS" regions (UCSC RefGene Group, *p* = 9.48e-20 and *p* = 7.28e-14, respectively). For gene and CpG level enrichment for the positive correlations see column 3 in Tables 3 and 4, respectively.

The differential methylation pattern distinguishing LumA-M1 from LumA-M3 samples could therefore be characterized by hundreds of CpGs that are hyper-methylated in the LumA-M1 samples. Distinct subsets of these CpGs correlate negatively and positively with the expression of developmental genes.

### Cox survival analysis

In previous sections, we presented two different partitions of luminal-A tumors based on genomic profiles, with prognostic value: The LumA-R2 group (characterized by high expression of immune-related genes) was associated with a reduced chance of 5-year recurrence, whereas the LumA-M1 group (characterized by hyper-methylation of CpGs located in developmental genes) was associated with poorer survival. To determine the prognostic contribution of the two partitions while adjusting for other relevant explanatory variables, we performed multivariate Cox survival analysis on both LumA-R and LumA-M partitions (see Table 5). Patients belonging to the LumA-M1 group had a 6.68-fold higher estimated 5-year death hazard compared with the other groups in the Cox multivariate model after adjustment for age, pathological stage, ER status, PR status and Her2 status. Patients belonging to the LumA-R2 group had a decreased recurrence hazard of 0.06 (that is, 94 % decrease) compared with LumA-R1 patients, after similar adjustment. The results reaffirm the independent prognostic value of the LumA-R2 and the LumA-M1 classes (see Additional file 1, section 10 for univariate analysis).

**Table 5:** Multivariate Cox analysis of luminal-A subgroups for five-year survival and five-year recurrence

	Survival		Recurrence	
Variable	Hazard ratio	*P* value	Hazard ratio	*P* value
LumA-R (1 vs 2)	0.56	0.36991	**0.06**	**0.00693**
LumA-M (2, 3 vs 1)	**6.68**	**0.00484**	3.04	0.07028
Age (<60 vs > =60 years)	**11.20**	**0.0037**	1.03	0.96530
Pathologic stage (I, II vs. III, IV)	2.12	0.25519	1.93	0.26992
ER status	7.17	0.18095	0.00	0.99575
PR status	0.47	0.50039	0.29	0.29092
Her2 status	1.48	0.72659	0.64	0.68789

Significant *p* values are marked in boldface. *ER* estrogen receptor, *PR* progesterone receptor, Her2 human epidermal growth factor receptor 2

## Discussion

Gene expression profiling has become a useful tool for breast cancer classification and for direction of treatment [59]. Although the HER2-enriched and the basal-like subgroups are well-defined and indicative for anti-Her2 and chemotherapy treatment, respectively, the ER-positive luminal subgroup still presents a clinical challenge. In general, all luminal tumors are candidates for anti-hormonal therapy. However, some tumors within this class, often with a more proliferative potential and conferring poorer outcome, are considered for additional therapy. Accordingly, the common classification based on the molecular intrinsic subtypes divides the luminal tumors into the luminal-A tumors, which have a better outcome, and the more proliferative luminal-B tumor subgroups, which have a worse outcome. However, this classification is sub-optimal for clinical decisions because the luminal tumors present a phenotypic and prognostic range rather than an exact partition to either group.

In this study, we applied unsupervised analysis on breast tumor samples using both expression and methylation profiles to reveal new genetic and epigenetic patterns that correlate with a clinical outcome, and compared them to the PAM50 subtypes. Overall, our analyses showed that the separation between luminal-A and luminal-B (as represented by PAM50 labels) is not clear-cut, but rather represents a phenotypic continuum (as previously observed [12, 60, 61]). In fact, each of the gene expression and methylation datasets used in our analysis separately enabled partitioning of the luminal samples into groups with better prognostic value than that of PAM50.

Furthermore, when we focused on the PAM50-designated luminal-A samples only, the RNA-Seq expression profiles split the luminal-A samples into two subgroups (Fig. 3a). The lobular-enriched LumA-R2 sample group, characterized by a distinct gene over-expression pattern, was associated with significantly reduced recurrence risk compared with the more proliferative LumA-R1 subgroup. Interestingly, genes constituting that over-expression pattern were significantly enriched for functions related to the immune system, including the more specific enrichment of chemokines and genes of upstream T cell receptor signaling pathways. We postulate that the significantly elevated mRNA levels of immune related genes in LumA-R2 samples are indicative of increased infiltration levels of immune system cells into these tumors.

Typically, chemokines serve as ligands that by binding to their corresponding receptors, attract immune system cells to the site where they are secreted [62, 63]. LumA-R2 samples over-expressed several chemokines and their corresponding receptors. The simultaneous over-expression of both the chemokine *CCL5* (previously found to be highly expressed by breast cancer cells [64]) and one of its receptors - *CCR5* (expressed among others by CD8+ cytotoxic T cells), suggests that tumor cell-derived *CCL5* attracts CD8+ cytotoxic T lymphocytes (CTLs) to LumA-R2 tumors. Similarly, the over-expressed chemokines *CCL19* and *CCL21* may be expressed by the tumor cells, whereas their *CCR7* receptor may be expressed by licensed DC or (less typically) by naive and central memory T cells.

In line with this possibility, the over-expressed genes in LumA-R2 samples included genes typical of CTLs (and also natural killer (NK) cells), which may lead to anti-tumor cytotoxic activities exerted by the granzyme (*GZMA* and *GZMB*) and perforin pathways (*PRF1*). Accordingly, over-expression of T cell activation genes was also detected in patients with LumA-R2 tumors. Notably, the over-expressed genes are concentrated at the upstream part of the T cell receptor-signaling pathway (Fig. 4). At this stage, it is not clear why downstream effectors are not enriched in LumA-R2 samples; however, it is of interest to see that the alpha chain of IL-15R was over-expressed in these samples, suggesting that T cell activation processes may indeed come into effect in this subgroup of patients.

How could the over-expression of the immune genes by LumA-R2 samples be related, if at all, to reduced tumor recurrence? It is possible that only LumA-R2 tumors can release chemoattractants that induce the migration of antigen-specific, possibly beneficial, leukocyte subpopulations to the tumor site. Despite recent reports associating tumor infiltrating lymphocytes with a better prognosis [65–67], it is yet to be determined how enhanced immunogenic activity in the LumA-R2 tumors may improve their outcome. Possibly in the future, this LumA-R2 characteristic pattern may direct emerging immune-checkpoint-related therapies [68].

The role of epigenetic regulation in malignant processes is increasingly recognized. Indeed, our analysis of DNA methylation data partitioned the breast tumor samples into four clusters showing only moderate agreement with the expression-based PAM50 subtypes. In line with previous studies [24, 69], one cluster had a hypo-methylation pattern and corresponded with the PAM50 basal-like subgroup that was associated with poorer outcome. However, the luminal samples did not cluster neatly into the PAM50 luminal-A and luminal-B subgroups. Instead, three luminal clusters with increasing methylation levels were obtained (clusters 1–3 in Fig. 5a), of which the most hyper-methylated cluster was associated with significantly poorer 5-year prognosis. In fact, even when we clustered only the luminal-A samples (Fig. 5c), the hyper-methylated cluster 1 (LumA-M1) was still associated with significantly poorer survival compared to the other two clusters (LumA-M2 and LumA-M3).

Notably, the top 1000 differentially methylated CpG loci, all hyper-methylated on LumA-M1 samples, had enrichment for genes involved in morphogenesis, differentiation, and developmental processes. Moreover, the CpG hyper-methylation correlated with under-expression of developmental genes, including various tumor suppressor genes. Indeed, hyper-methylation of developmental genes in luminal breast tumors was previously reported [70, 71], secondary to repressive histone marks, which direct *de novo* methylation. Moreover, hyper-methylation was implicated in normal processes of cell aging and in tumorigenesis [61]. Taken together, the methylation-based analysis suggests a poorer outcome for luminal tumors with a characteristic hyper-methylation pattern, whether in the luminal-A or in the luminal-B subgroups. The hyper-methylation-associated silencing of developmental and tumor suppressor genes may indeed explain these findings. More importantly, within the luminal-A subgroup that is generally associated with a better outcome, the hyper-methylation pattern of the LumA-M1 subgroup marks 84 samples (comprising 22 % of the 378 luminal-A samples) as a high-risk patient group that might benefit from more aggressive treatment.

Last, we showed that the sample partitions induced by the gene expression and DNA methylation patterns are related (*p* = 4.4e-08; see lower bar in Fig. 5c), mainly because the LumA-M3 samples that are associated with a better outcome are enriched for LumA-R2. However, our attempts to partition the luminal-A samples based on both patterns together did not yield a partition that is better than the separate partitions, in terms of survival prediction or clustering stability. This observation was confirmed by Cox multivariate analysis showing the independent prognostic contribution of each pattern to outcome prediction (Table 5), suggesting that gene expression and methylation hold complementary information, reflecting different aspects of the biological complexity of breast tumors.

Very recently, several novel partitions of luminal breast tumors were proposed [19, 65, 72]. The partitions identified in this study are reinforced by partial though significant similarity to some newly defined groups. LumA-R1 and LumA-R2 clusters are enriched for the proliferative (*p* = 8.1e-04) and reactive-like (2.4-e04) classes of invasive lobular carcinoma (ILC), respectively, as defined in [73] (see Additional file 1, section 12). Furthermore, the LumA-M1 cluster is enriched (*p* = 1.6e-07) for the Epi-LumB group of tumors that are associated with poorer outcome, described by Stefansson et al. [69] (named Epi-LumB, as it was largely composed of Luminal-B samples, see Additional file 1, section 13). Additional research is needed in order to consolidate the different partitions identified using different procedures into robust and meaningful categories for prognostic and diagnostic use in clinics.

## Conclusions

This study emphasizes the large heterogeneity of luminal breast tumors in general, and of luminal-A samples in particular, the inner variability of which was found to be inadequately captured by PAM50 molecular subtypes. Analysis of the RNA-Seq data revealed a partition of the luminal-A samples into groups associated with different risks of 5-year recurrence. We suggest that the over-expression of immune genes in the LumA-R2 group can be ascribed to a higher tendency of its samples to attract tumor-infiltrating lymphocytes, but this requires further research into the mechanism by which the higher infiltrates affect recurrence risk. In the DNA methylation data, a hyper-methylation pattern enriched for developmental genes defined a luminal-A subgroup that was associated with poorer patient survival. In practice, the two prognostic patterns and the lists of genomic features characterizing each of them, can uncover the biological aspects underlying the heterogeneity of luminal-A tumors, improve our ability to classify these tumors into more accurate clinical subgroups, and contribute to the development of novel directed therapies.

## Abbreviations

CTL, cytotoxic T lymphocytes; DMC, differentially methylated CpG; DMR, differentially methylated region; ER, estrogen receptor; FDR, false discovery rate; GO, Gene Ontology; HER2, human epidermal growth factor 2; IL, interleukin; KEGG, Kyoto Encyclopedia of Genes and Genomes; NK, natural killer; PR, progesterone receptor; TCGA, The Cancer Genome Atlas; TCR, T cell receptor; TNF, tumor necrosis factor

## Additional files

Additional file 1: Supplementary Information. (DOCX 20306 kb) Additional file 2: Detailed cohort description - TCGA sample IDs for the 6 sample groups analyzed in the study. (XLSX 179 kb) Additional file 3: LumA-R1 vs R2 analysis results. List of the 1000 top differentially expressed genes. Enrichment analysis results. (XLSX 974 kb) Additional file 4: LumA-M1 vs M3 analysis results. List of the top 1000 differentially methylated CpGs. Enrichment analysis results. (XLSX 242 kb) Additional file 5: LumA-M1 vs M3 analysis results (negatively correlated to expression only). List of differentially methylated CpGs that are negatively correlated to expression. Enrichment analysis results. (XLSX 16141 kb) Additional file 6: LumA-M1 vs M3 analysis results (positively correlated to expression only). List of differentially methylated CpGs that are positively correlated to expression. Enrichment analysis results. (XLSX 16148 kb)
