# Erratum to: Expression and methylation patterns partition luminal-A breast tumors into distinct prognostic subgroups

**DOI:** 10.1186/s13058-016-0775-4

**Published:** 2016-11-28

**Authors:** Dvir Netanely, Ayelet Avraham, Adit Ben-Baruch, Ella Evron, Ron Shamir

**Affiliations:** 1 Blavatnik School of Computer Science, Tel Aviv University, Tel Aviv, Israel; 2 Oncology Department, Assaf Harofeh Medical Center, Tsrifin, Israel; 3 Department of Cell Research and Immunology, George S. Wise Faculty of Life Sciences, Tel Aviv University, Tel Aviv, Israel

## Erratum

After publication of this article [1], the authors requested to add an official acknowledgement for using the TCGA Breast Cancer dataset in their article. The following information has therefore been added to the Acknowledgements section: “The results published here are based upon data generated by The Cancer Genome Atlas managed by the NCI and NHGRI. Information about TCGA can be found at http://cancergenome.nih.gov“. The amended Acknowledgements section has been included in this erratum accordingly.
