# Supplementary material for: Expression and methylation patterns partition luminal-A breast tumors into distinct prognostic subgroups
Source: Breast Cancer Res. 2016 Jul 7;18:74. doi: 10.1186/s13058-016-0724-2 (PMC4936004; doi:10.1186/s13058-016-0724-2)
Supplement: Additional file 1: — Supplementary Information. (DOCX 20306 kb) [file 13058_2016_724_MOESM1_ESM.docx]

**Expression and methylation patterns partition Luminal-A breast tumors into distinct prognostic subgroups**

Dvir Netanely^a^, Ayelet Avraham^b^, Adit Ben-Baruch^c^, Ella Evron^b^, Ron Shamir^a^

^a^Blavatnik School of Computer Science, Tel Aviv University, Tel Aviv, Israel

^b^Oncology Department, Assaf Harofeh Medical Center, Tsrifin, Israel

^c^Department of Cell Research and Immunology, George S. Wise Faculty of Life Sciences, Tel Aviv University, Tel Aviv, Israel

**Additional file 1**

**Datasets**

TCGA's Breast Cancer datasets were downloaded from UCSC cancer browser website on March 2015.

| Technology | Dataset title | DatasetID | #Samples | Dataset version |
| --- | --- | --- | --- | --- |
| Gene Expression - RNA-Seq | TCGA breast invasive carcinoma (BRCA) gene expression by RNAseq (IlluminaHiSeq) | BRCA gene expression (IlluminaHiSeq) | 1215 | 2015-02-24 |
| DNA-Methylation array | TCGA breast invasive carcinoma (BRCA) (HumanMethylation450) | BRCA (Methylation450k) | 872 | 2015-02-24 |
| Gene Expression - MicroArrays | TCGA breast invasive carcinoma (BRCA) gene expression (AgilentG4502A_07_3 array) | BRCA gene expression (AgilentG4502A_07_3) | 597 | 2015-02-24 |

**Table S1-1** Properties of datasets used in the study

1. Global RNA-Seq dataset analysis (Normal + Tumor)

**Obtaining the RNA-Seq dataset and initial sample preprocessing**

RSEM normalized version of TCGA's BRCA RNA-SEQ expression dataset was used in the following analyses.

Updated RNA-SEQ based PAM50 calls for TCGA BRCA samples were obtained from UNC University.

Sample preprocessing: Downloaded dataset contained 1215 samples of which the following were removed based on supplied labels: 19 – Unknown tissue site, 11 Male, 7 metastatic sample, 30 Unavailable UNC_Pam50 labels. Preprocessed dataset contained 1148 samples, of which 113 are normal based on the 'sample type' field, and 150 are normal based on 'PAM50 call'.

**Distribution of PAM50 calls on the preprocessed RNA-Seq expression dataset**

Total after preprocessing: 1148

| Basal | 183 |
| --- | --- |
| Her2 | 78 |
| LumA | 534 |
| LumB | 203 |
| Normal | 150 |

**Figure S-1A:** PCA of 1148 breast samples based on 2000 top variable genes, colored by PAM50 labels

**Clustering the samples based on RNA-SEQ data**

The K-Means clustering algorithm was executed on the 1148 samples using the 2000 top variable genes. Matlab v8.5 implementation of the K-Means algorithm was used using correlation based distance metric, and 100 replicates. Rows (genes) were standardized before the sample clustering.

**Figure S-1B:** PCA of 1148 breast samples based on 2000 top variable genes, colored by K-Means clusters.

|  | **RNA-Seq Clusters** | **Total** | **1** | **2** | **3** | **4** | **5** |
| --- | --- | --- | --- | --- | --- | --- | --- |
|  |  | **n=1148** | **n=360** | **n=217** | **n=201** | **n=193** | **n=177** |
| **Age (Median)** |  | 58 | 62 | 57 | 58 | 53 | 54 |
| **ER Status** | NA | 160 ( 14%) | 28 ( 8%) | 2 ( 1%) | 8 ( 4%) | 7 ( 4%) | 115 ( 65%) |
|  | Negative | 227 ( 20%) | 3 ( 1%) | 7 ( 3%) | 53 ( 26%) | 159 ( 82%) | 5 ( 3%) |
|  | Positive | 761 ( 66%) | 329 ( 91%) | 208 ( 96%) | 140 ( 70%) | 27 ( 14%) | 57 ( 32%) |
| **PR Status** | NA | 163 ( 14%) | 29 ( 8%) | 4 ( 2%) | 6 ( 3%) | 9 ( 5%) | 115 ( 65%) |
|  | Negative | 326 ( 28%) | 35 ( 10%) | 21 ( 10%) | 86 ( 43%) | 171 ( 89%) | 13 ( 7%) |
|  | Positive | 659 ( 57%) | 296 ( 82%) | 192 ( 88%) | 109 ( 54%) | 13 ( 7%) | 49 ( 28%) |
| **Her2 Status** | NA | 391 ( 34%) | 81 ( 23%) | 73 ( 34%) | 37 ( 18%) | 57 ( 30%) | 143 ( 81%) |
|  | Negative | 649 ( 57%) | 260 ( 72%) | 137 ( 63%) | 89 ( 44%) | 132 ( 68%) | 31 ( 18%) |
|  | Positive | 108 ( 9%) | 19 ( 5%) | 7 ( 3%) | 75 ( 37%) | 4 ( 2%) | 3 ( 2%) |
| **PAM50** | Basal | 183 ( 16%) | 0 ( 0%) | 0 ( 0%) | 3 ( 1%) | 180 ( 93%) | 0 ( 0%) |
|  | Her2 | 78 ( 7%) | 0 ( 0%) | 0 ( 0%) | 78 ( 39%) | 0 ( 0%) | 0 ( 0%) |
|  | LumA | 534 ( 47%) | 242 ( 67%) | 212 ( 98%) | 37 ( 18%) | 0 ( 0%) | 43 ( 24%) |
|  | LumB | 203 ( 18%) | 117 ( 33%) | 5 ( 2%) | 80 ( 40%) | 1 ( 1%) | 0 ( 0%) |
|  | Normal | 150 ( 13%) | 1 ( 0%) | 0 ( 0%) | 3 ( 1%) | 12 ( 6%) | 134 ( 76%) |
| **Pathologic stage** | NA | 120 ( 10%) | 1 ( 0%) | 2 ( 1%) | 2 ( 1%) | 2 ( 1%) | 113 ( 64%) |
|  | Stage I | 176 ( 15%) | 64 ( 18%) | 53 ( 24%) | 18 ( 9%) | 29 ( 15%) | 12 ( 7%) |
|  | Stage II | 589 ( 51%) | 202 ( 56%) | 108 ( 50%) | 118 ( 59%) | 134 ( 69%) | 27 ( 15%) |
|  | Stage III | 234 ( 20%) | 81 ( 23%) | 49 ( 23%) | 58 ( 29%) | 23 ( 12%) | 23 ( 13%) |
|  | Stage IV | 16 ( 1%) | 5 ( 1%) | 1 ( 0%) | 5 ( 2%) | 4 ( 2%) | 1 ( 1%) |
|  | Stage X | 13 ( 1%) | 7 ( 2%) | 4 ( 2%) | 0 ( 0%) | 1 ( 1%) | 1 ( 1%) |
| **Histological type** | Infiltrating Ductal Carcinoma | 753 ( 66%) | 272 ( 76%) | 107 ( 49%) | 182 ( 91%) | 166 ( 86%) | 26 ( 15%) |
|  | Infiltrating Lobular Carcinoma | 182 ( 16%) | 40 ( 11%) | 95 ( 44%) | 11 ( 5%) | 1 ( 1%) | 35 ( 20%) |
|  | Medullary Carcinoma | 5 ( 0%) | 0 ( 0%) | 0 ( 0%) | 1 ( 0%) | 4 ( 2%) | 0 ( 0%) |
|  | Metaplastic Carcinoma | 4 ( 0%) | 0 ( 0%) | 0 ( 0%) | 1 ( 0%) | 3 ( 2%) | 0 ( 0%) |
|  | Mixed Histology | 29 ( 3%) | 15 ( 4%) | 8 ( 4%) | 3 ( 1%) | 1 ( 1%) | 2 ( 1%) |
|  | Mucinous Carcinoma | 16 ( 1%) | 15 ( 4%) | 0 ( 0%) | 1 ( 0%) | 0 ( 0%) | 0 ( 0%) |
|  | NA | 159 ( 14%) | 18 ( 5%) | 7 ( 3%) | 2 ( 1%) | 18 ( 9%) | 114 ( 64%) |

**Table S-1A:** Cohort description for the global RNA-Seq dataset analysis (Normal + Tumor)

**Comparing resulting clusters to PAM50 labels**

**Figure S-1C:** Distribution of PAM50 labels among sample clusters

**Figure S-1D:** pValues of the hyper geometric enrichment of resulting clusters for PAM50 labels

**Evaluation of expression distribution in cluster 1 samples versus cluster 2 samples**

**Figure S-1E:** Distribution of normalized expression values by sample cluster.

When applying rank sum test on the top 2000 variables genes, testing for difference in means between samples of cluster 1 (n=360) and samples of cluster 2(n=217), 1421 genes out of the 2000 passed the test with pValue<0.01.

| All genes passing the test | 1421 |
| --- | --- |
| Genes over expressed on cluster1 compared to cluster2 | 229 |
| Genes over expressed on cluster2 compared to cluster1 | 1184 |
| Genes with FC==0 | 8 |

**Table S-1B:** Analysis of differentially expressed genes

**Figure S-1F:** Differentially expressed genes between cluster 1 and cluster 2

1. **RNA-Seq Luminal samples analysis**

Zooming in to the Luminal samples, we applied unsupervised analysis only on samples labeled as either Luminal-A or Luminal-B by PAM50.

Sample preprocessing: In this step of the analysis we started with the 1215 samples included in the TCGA's BRCA RNA-Seq dataset and removed the following samples: 19 – Unknown tissue site, 11 Male, 7 metastatic, 30 Unavailable PAM50 labels, 113 normal sample type, 37 normal by PAM50. From the Remaining with 988 samples we kept only the 737 Luminal samples (534 Luminal-A and 203 Luminal-B based on PAM50 labels).

Gene preprocessing: We kept only the top 2000 variable genes over the 737 Luminal samples.

Unsupervised method: As described in the previous section, K-Means (distance metric: correlation) with K=2 applied on the 737 samples using the 2000 top variable genes (after row standardization).

We then compared the sample partition induced by our clustering, to the PAM50 Luminal-A/Luminal-B partition using log rank tests and show that our RNA-Seq based partition outperforms PAM50's partition in terms of both survival and recurrence, and in both 5-year and overall time spans.

**OVERALL Survival and Recurrence**

|  | RNA-SEQ Clusters | PAM50's LuminalA-LuminalB |
| --- | --- | --- |
| OVERALL SURVIVAL | ******  pValue=0.0029 |   pValue=0.0057 |
| OVERALL RECURRENCE |   pValue = 0.028 |   pValue = 0.990 |

**Figure S-2A:** Overall survival and recurrence plots for K-Means clusters versus PAM50 Luminal-A/Luminal-B classification

|  | **RNA-Seq Clusters** | **Total** | **1** | **2** |
| --- | --- | --- | --- | --- |
|  |  | n=737 | n=382 | n=355 |
| **Age (Median)** |  | 60 | 62 | 56 |
| **ER Status** | NA | 33 ( 4%) | 27 ( 7%) | 6 ( 2%) |
|  | Negative | 14 ( 2%) | 4 ( 1%) | 10 ( 3%) |
|  | Positive | 690 ( 94%) | 351 ( 92%) | 339 ( 95%) |
| **PR Status** | NA | 36 ( 5%) | 28 ( 7%) | 8 ( 2%) |
|  | Negative | 87 ( 12%) | 44 ( 12%) | 43 ( 12%) |
|  | Positive | 614 ( 83%) | 310 ( 81%) | 304 ( 86%) |
| **Her2 Status** | NA | 195 ( 26%) | 77 ( 20%) | 118 ( 33%) |
|  | Negative | 486 ( 66%) | 270 ( 71%) | 216 ( 61%) |
|  | Positive | 56 ( 8%) | 35 ( 9%) | 21 ( 6%) |
| **PAM50** | LumA | 534 ( 72%) | 207 ( 54%) | 327 ( 92%) |
|  | LumB | 203 ( 28%) | 175 ( 46%) | 28 ( 8%) |
| **Pathologic stage** | NA | 3 ( 0%) | 1 ( 0%) | 2 ( 1%) |
|  | Stage I | 137 ( 19%) | 54 ( 14%) | 83 ( 23%) |
|  | Stage II | 396 ( 54%) | 224 ( 59%) | 172 ( 48%) |
|  | Stage III | 179 ( 24%) | 90 ( 24%) | 89 ( 25%) |
|  | Stage IV | 10 ( 1%) | 6 ( 2%) | 4 ( 1%) |
|  | Stage X | 12 ( 2%) | 7 ( 2%) | 5 ( 1%) |
| **Histological type** | Infiltrating Ductal Carcinoma | 504 ( 68%) | 307 ( 80%) | 197 ( 55%) |
|  | Infiltrating Lobular Carcinoma | 163 ( 22%) | 29 ( 8%) | 134 ( 38%) |
|  | Medullary Carcinoma | 1 ( 0%) | 0 ( 0%) | 1 ( 0%) |
|  | Mixed Histology | 27 ( 4%) | 15 ( 4%) | 12 ( 3%) |
|  | Mucinous Carcinoma | 16 ( 2%) | 15 ( 4%) | 1 ( 0%) |
|  | NA | 26 ( 4%) | 16 ( 4%) | 10 ( 3%) |

**Table S-2A:** Cohort description for the Luminal RNA-Seq dataset analysis

1. **RNA-Seq Luminal-A sample analysis**

Same as previous section, but removed the 203 Luminal-B samples based on PAM50 labels as well.

Remained with 534 Luminal-A samples.

K-Means (distance metric: correlation) with K=2 applied on the 534 samples using the 2000 top variable genes.

|  | **RNA-Seq Clusters** | **Total** | **1** | **2** |
| --- | --- | --- | --- | --- |
|  |  | n=534 | n=258 | n=276 |
| **Age (Median)** |  | 60 | 62 | 57 |
| **ER Status** | NA | 21 ( 4%) | 18 ( 7%) | 3 ( 1%) |
|  | Negative | 11 ( 2%) | 3 ( 1%) | 8 ( 3%) |
|  | Positive | 502 ( 94%) | 237 ( 92%) | 265 ( 96%) |
| **PR Status** | NA | 24 ( 4%) | 19 ( 7%) | 5 ( 2%) |
|  | Negative | 50 ( 9%) | 21 ( 8%) | 29 ( 11%) |
|  | Positive | 460 ( 86%) | 218 ( 84%) | 242 ( 88%) |
| **Her2 Status** | NA | 161 ( 30%) | 62 ( 24%) | 99 ( 36%) |
|  | Negative | 347 ( 65%) | 181 ( 70%) | 166 ( 60%) |
|  | Positive | 26 ( 5%) | 15 ( 6%) | 11 ( 4%) |
| **PAM50** | LumA | 534 (100%) | 258 (100%) | 276 (100%) |
| **Pathologic stage** | NA | 3 ( 1%) | 1 ( 0%) | 2 ( 1%) |
|  | Stage I | 113 ( 21%) | 52 ( 20%) | 61 ( 22%) |
|  | Stage II | 282 ( 53%) | 144 ( 56%) | 138 ( 50%) |
|  | Stage III | 121 ( 23%) | 52 ( 20%) | 69 ( 25%) |
|  | Stage IV | 6 ( 1%) | 4 ( 2%) | 2 ( 1%) |
|  | Stage X | 9 ( 2%) | 5 ( 2%) | 4 ( 1%) |
| **Histological type** | Infiltrating Ductal Carcinoma | 331 ( 62%) | 188 ( 73%) | 143 ( 52%) |
|  | Infiltrating Lobular Carcinoma | 152 ( 28%) | 35 ( 14%) | 117 ( 42%) |
|  | Mixed Histology | 21 ( 4%) | 12 ( 5%) | 9 ( 3%) |
|  | Mucinous Carcinoma | 11 ( 2%) | 10 ( 4%) | 1 ( 0%) |
|  | NA | 19 ( 4%) | 13 ( 5%) | 6 ( 2%) |

**Table S-3A:** Cohort description for the Luminal-A RNA-Seq dataset analysis

|  | **SURVIVAL** | **RECURRENCE** |
| --- | --- | --- |
| **5 YEAR** | **** |  |
| **OVERALL** | **** | **** |

**Figure S-3A:** Survival and Recurrence analysis for the 2 Luminal–A subgroups

**Cluster LumA-R2 samples exhibit distinct overexpression pattern**

Applied rank sum test on the top 2000 variables genes, testing for difference in means between samples of LumA-R1 (n=258) and LumA-R2 samples (n=276).

1276 genes out of the 2000 passed the test with pValue<0.01.

| Total number of genes passing the rank sum test | 1276 |
| --- | --- |
| Genes over expressed on cluster1 compared to cluster2 | 194 |
| Genes over expressed on cluster2 compared to cluster1 | 1068 |
| Genes with zero fold change | 5 |

**Table S-3B:** Analysis of differentially expressed genes

1. **Validation of Luminal-A partition on Microarray gene expression data**

In order to verify that the two Luminal-A subgroups that were identified using the RNA-Seq data represent real biological variance rather than measurement or normalization bias, we repeated the analysis on microarray based gene expression data.

TCGA's Microarray gene expression data was downloaded from the Cancer Browser website. Original dataset contained 597 samples x 17814 genes. We removed 11 samples (6 Male, 3 Metastatic and 2 having unknown tissue site) and remained with 586 samples.

Samples were clustered using the same protocol described for the RNA-Seq dataset (K-means algorithm applied using correlation distance after row normalization). Similarly to the Global RNA-Seq analysis. Luminal-A samples were split between a mixed Luminal-A/Luminal-B cluster (cluster 1) and a rather homogenous cluster (cluster 2).

**Figure S-4A** Global unsupervised clustering of breast samples using Microarray gene expression data.

When clustering the 265 Luminal-A samples in the microarray dataset into 2, the resulting partition exhibited very high similarity (Chi-square p=1.1e-40) to the Luminal-A subgroups identified based on the RNA-Seq data. When comparing the top 200 genes differentially expressed on the two-microarray Luminal-A subgroups to the top 200 genes differentially expressed on the two RNA-Seq Luminal-A subgroups, 88 genes appeared in the intersection. Similarly, to the RNA-Seq based list of differentially expressed genes, the 88 genes also were enriched for GO terms such as immune system process, cell differentiation and T-Cell receptor related terms.

We therefore conclude that the signal observed on the RNA-Seq data, splitting the Luminal-A samples into two distinct subgroups is not an artifact of either the measurement technology or the normalization used by TCGA.

1. **Differentially Expressed Gene Analysis (LumA-R1 vs. LumA-R2)**
   1. **Gene Enrichment tests on the top 1000 differentially expressed genes**

We started our analysis of differentially expressed genes between the two subgroups identified using RNA-Seq data within Luminal-A samples, by generating a list of the top 1000 DEGs using the rank sum test pValue, and a requirement for a minimum mean difference of 0.5. Interestingly, all 1000 genes were overexpressed in LumA-R2 compared to LumA-R1.

We then used the Expandar[1] suite to detect significant enrichments for Gene ontology terms[2], KEGG pathways[3] and Wiki-pathways[4]. The results are listed below:

**Gene Ontology enrichments detected using Expander TANGO on the list of 1000 DEGs**

| **Gene Ontology Term** | **#Genes** | **Enrichment significance (pValue)** | **TANGO corrected pvalue** |
| --- | --- | --- | --- |
| regulation of immune system process - GO:0002682 | 152 | 3.74E-50 | 1.00E-05 |
| immune system process - GO:0002376 | 201 | 3.65E-47 | 1.00E-05 |
| regulation of leukocyte activation - GO:0002694 | 71 | 2.37E-28 | 1.00E-05 |
| regulation of multicellular organismal process - GO:0051239 | 183 | 2.89E-28 | 1.00E-05 |
| cell activation - GO:0001775 | 91 | 4.59E-28 | 1.00E-05 |
| regulation of response to external stimulus - GO:0032101 | 73 | 8.18E-27 | 1.00E-05 |
| regulation of biological quality - GO:0065008 | 218 | 1.82E-26 | 1.00E-05 |
| leukocyte activation - GO:0045321 | 67 | 1.95E-26 | 1.00E-05 |
| positive regulation of cell activation - GO:0050867 | 56 | 5.13E-24 | 1.00E-05 |
| T cell activation - GO:0042110 | 45 | 4.93E-22 | 1.00E-05 |
| regulation of cell proliferation - GO:0042127 | 128 | 1.83E-21 | 1.00E-05 |
| regulation of response to stress - GO:0080134 | 91 | 1.91E-19 | 1.00E-05 |
| chemical homeostasis - GO:0048878 | 93 | 6.50E-19 | 1.00E-05 |
| hemopoiesis - GO:0030097 | 60 | 6.63E-19 | 1.00E-05 |
| cell migration - GO:0016477 | 79 | 9.97E-19 | 1.00E-05 |
| locomotion - GO:0040011 | 110 | 1.13E-18 | 1.00E-05 |
| immune response-regulating cell surface receptor signaling pathway - GO:0002768 | 36 | 2.88E-18 | 1.00E-05 |
| lymphocyte differentiation - GO:0030098 | 37 | 3.09E-18 | 1.00E-05 |
| leukocyte migration - GO:0050900 | 43 | 1.10E-17 | 1.00E-05 |
| regulation of cytokine production - GO:0001817 | 58 | 8.45E-17 | 1.00E-05 |
| positive regulation of cell proliferation - GO:0008284 | 79 | 1.46E-16 | 1.00E-05 |
| cell differentiation - GO:0030154 | 194 | 2.08E-16 | 1.00E-05 |
| biological adhesion - GO:0022610 | 90 | 6.97E-16 | 1.00E-05 |
| response to organic substance - GO:0010033 | 155 | 8.13E-16 | 1.00E-05 |
| calcium ion homeostasis - GO:0055074 | 43 | 1.11E-15 | 1.00E-05 |
| cellular response to cytokine stimulus - GO:0071345 | 60 | 2.42E-15 | 1.00E-05 |
| cellular response to chemical stimulus - GO:0070887 | 137 | 3.04E-15 | 1.00E-05 |
| immune effector process - GO:0002252 | 41 | 1.72E-14 | 1.00E-05 |
| regulation of cell migration - GO:0030334 | 55 | 3.10E-14 | 1.00E-05 |
| regulation of acute inflammatory response - GO:0002673 | 20 | 4.06E-14 | 1.00E-05 |
| hemostasis - GO:0007599 | 62 | 4.15E-13 | 1.00E-05 |
| negative regulation of biological process - GO:0048519 | 213 | 1.29E-12 | 1.00E-05 |
| positive regulation of signaling - GO:0023056 | 83 | 1.55E-12 | 1.00E-05 |
| response to external stimulus - GO:0009605 | 104 | 2.20E-12 | 1.00E-05 |
| blood circulation - GO:0008015 | 39 | 9.70E-12 | 1.00E-05 |
| regulation of behavior - GO:0050795 | 28 | 1.21E-11 | 1.00E-05 |
| positive regulation of cellular component movement - GO:0051272 | 37 | 1.54E-11 | 1.00E-05 |
| regulation of adaptive immune response - GO:0002819 | 23 | 2.43E-11 | 1.00E-05 |
| regulation of cell differentiation - GO:0045595 | 89 | 3.88E-11 | 1.00E-05 |
| negative regulation of sequestering of calcium ion - GO:0051283 | 13 | 5.22E-11 | 1.00E-05 |
| regulation of cell death - GO:0010941 | 107 | 5.51E-11 | 1.00E-05 |
| regulation of secretion - GO:0051046 | 53 | 5.55E-11 | 1.00E-05 |
| regulation of alpha-beta T cell activation - GO:0046634 | 18 | 9.86E-11 | 1.00E-05 |
| regulation of hydrolase activity - GO:0051336 | 89 | 1.31E-10 | 1.00E-05 |
| regulation of protein secretion - GO:0050708 | 25 | 1.54E-10 | 1.00E-05 |
| humoral immune response - GO:0006959 | 22 | 2.04E-10 | 1.00E-05 |
| positive regulation of inflammatory response - GO:0050729 | 19 | 2.82E-10 | 1.00E-05 |
| regulation of lymphocyte mediated immunity - GO:0002706 | 19 | 1.28E-09 | 1.00E-05 |
| cell chemotaxis - GO:0060326 | 21 | 3.53E-09 | 1.00E-05 |
| regulation of protein transport - GO:0051223 | 36 | 3.98E-09 | 1.00E-05 |
| positive regulation of metabolic process - GO:0009893 | 144 | 5.02E-09 | 1.00E-05 |
| regulation of leukocyte chemotaxis - GO:0002688 | 16 | 5.52E-09 | 1.00E-05 |
| vasculature development - GO:0001944 | 47 | 7.58E-09 | 1.00E-05 |
| nervous system development - GO:0007399 | 127 | 7.73E-09 | 1.00E-05 |
| positive regulation of leukocyte migration - GO:0002687 | 16 | 9.39E-09 | 1.00E-05 |
| regulation of transmembrane transport - GO:0034762 | 45 | 1.00E-08 | 1.00E-05 |
| positive regulation of molecular function - GO:0044093 | 104 | 1.03E-08 | 1.00E-05 |
| negative regulation of multicellular organismal process - GO:0051241 | 41 | 1.11E-08 | 1.00E-05 |

**KEGG PATHWAYS**

| **KEGG Pathway** | **#Genes** | **p-value** | **Enrichment** | **Genes** |
| --- | --- | --- | --- | --- |
| Cytokine-cytokine receptor interaction | 56 | 4.76E-22 | 4.57 | [ACVRL1, CD40, CXCL9, TNFRSF13B, CXCL2, CX3CL1, IL18RAP, LEPR, TNFRSF8, IL12B, CCR7, CCR5, CCR4, CCR2, PDGFRA, IL15RA, IL11RA, IL1R2, TNFRSF1B, TGFBR2, IL3RA, KIT, XCL2, XCL1, LTB, MET, CCL14, CCL13, FIGF, CXCR5, CSF2RB, CXCR6, IL2RG, EGFR, TPO, CCL5, CXCR3, TNFRSF17, CCL19, IL12RB1, CCL17, NGFR, CCL23, XCR1, CCL21, TSLP, IL10RA, IL6, BMP2, CXCL12, CD40LG, LEP, FAS, CD27, IL7R, IL18R1] |
| Hematopoietic cell lineage | 29 | 1.50E-17 | 7.1 | [CD1E, CD3G, CD1D, CD1C, CD3E, CD1B, CD3D, TPO, CD19, CD38, CD37, CD36, CD34, CR2, CR1, MME, IL11RA, IL1R2, CD2, FCER2, IL6, CD8B, CD5, CD8A, IL3RA, CD7, KIT, IL7R, MS4A1] |
| Cell adhesion molecules (CAMs) | 30 | 4.08E-13 | 4.84 | [CD40, ICAM2, NRXN2, SPN, CDH5, HLA-DOA, CD34, HLA-DOB, JAM2, JAM3, CADM3, PDCD1LG2, SELE, HLA-E, CD2, SELP, CLDN11, CLDN5, PTPRC, CD40LG, CD6, CD8B, SELL, CD8A, HLA-DPB1, CLDN19, PECAM1, CD28, CD226, PDCD1] |
| Primary immunodeficiency | 16 | 8.70E-13 | 9.73 | [CD40, CIITA, TNFRSF13B, IL2RG, CD3E, CD3D, CD79A, ZAP70, CD40LG, PTPRC, CD8B, LCK, CD8A, CD19, IL7R, JAK3] |
| Chemokine signaling pathway | 31 | 1.14E-09 | 3.49 | [CCL14, CCL13, ITK, CXCL9, CXCR5, ADCY4, PIK3CD, CXCR6, RASGRP2, CXCL2, CX3CL1, GNG2, CCL5, CXCR3, CCR7, CCL19, CCR5, CCL17, CCR4, JAK3, CCR2, CCL23, XCR1, CCL21, PRKCB, GNG11, FGR, CXCL12, ELMO1, XCL2, XCL1] |
| Complement and coagulation cascades | 17 | 1.36E-08 | 5.24 | [CR2, CR1, C1S, VWF, F10, CFH, C1R, PROS1, F2R, CFI, TFPI, C3, C6, C7, SERPING1, MASP1, A2M] |
| T cell receptor signaling pathway | 20 | 1.30E-07 | 3.94 | [ITK, PIK3CD, CD3G, CD3E, CD3D, ZAP70, PTPRC, CD40LG, CD8B, CD8A, LCK, GRAP2, CD28, PAK7, PRKCQ, FYN, CD247, PDCD1, PAK3, LAT] |
| Allograft rejection | 11 | 6.44E-07 | 6.33 | [CD40, CD40LG, HLA-DPB1, PRF1, CD28, GZMB, FAS, IL12B, HLA-DOA, HLA-DOB, HLA-E] |
| Natural killer cell mediated cytotoxicity | 20 | 5.66E-06 | 3.13 | [PRKCB, SH2D1A, PRF1, ICAM2, GZMB, PIK3CD, PRKCA, HLA-E, ZAP70, NCR3, KLRK1, LCK, PLCG2, FAS, CD48, FYN, CD247, HCST, LAT, CD244] |
| Pathways in cancer | 34 | 1.49E-05 | 2.2 | [FIGF, LAMA2, EPAS1, TCF7, PIK3CD, PTGS2, GLI1, ETS1, FGF2, FOXO1, EGFR, GLI2, WNT6, FGF7, ACVR1C, MECOM, PLCG2, WNT1, RUNX1T1, PDGFRA, WNT10A, PRKCB, PTCH2, PRKCA, IGF1, TRAF1, TGFBR2, IL6, BMP2, COL4A4, KIT, FAS, PPARG, MET] |
| PPAR signaling pathway | 13 | 1.67E-05 | 4.01 | [ADIPOQ, LPL, AQP7, ACSL5, ACSL4, FABP4, ACADL, FABP7, PPARG, PLIN1, CD36, PCK1, PLTP] |
| Autoimmune thyroid disease | 11 | 2.39E-05 | 4.5 | [CD40, TPO, CD40LG, HLA-DPB1, PRF1, CD28, GZMB, FAS, HLA-DOA, HLA-DOB, HLA-E] |
| Focal adhesion | 23 | 6.43E-05 | 2.46 | [PDGFRA, FIGF, TNXB, VWF, LAMA2, CAV2, PRKCB, CAV1, PIK3CD, PRKCA, IGF1, EGFR, THBS4, RELN, TNN, COL4A4, PAK7, ITGA7, COL6A6, FYN, FLNC, PAK3, MET] |
| Neuroactive ligand-receptor interaction | 27 | 7.73E-05 | 2.25 | [PTGER4, PTGFR, HTR2B, ADRB2, HTR2A, P2RY8, EDNRB, CNR2, GRM7, CNR1, S1PR1, LEPR, CTSG, S1PR2, GABRE, S1PR4, GRIA4, GABRP, GZMA, P2RY14, F2R, AVPR2, SSTR1, TACR1, P2RX1, LEP, F2RL2] |
| Calcium signaling pathway | 21 | 9.04E-05 | 2.53 | [PDGFRA, PTGFR, PDE1B, PRKCB, PDE1A, F2R, HTR2B, ADCY4, PRKCA, HTR2A, TACR1, ADRB2, RYR3, EGFR, SLC8A3, EDNRB, GNAL, PHKG1, P2RX1, PLCG2, CD38] |
| Graft-versus-host disease | 9 | 9.67E-05 | 4.67 | [IL6, HLA-DPB1, PRF1, CD28, GZMB, FAS, HLA-DOA, HLA-DOB, HLA-E] |

**WIKI-PATHWAYS**

| **Wiki-Pathway** | **#Genes** | **p-value** | **Enrichment** | **Genes** |
| --- | --- | --- | --- | --- |
| TCR Signaling Pathway | 10 | 1.55E-09 | 11.8 | [IL15RA, ITK, PSTPIP1, CD8A, GRAP2, CD3G, CD247, CD3E, CD3D, LAT] |
| B Cell Receptor Signaling Pathway | 10 | 1.72E-06 | 6.45 | [MAP4K1, BLK, KLF11, CR2, PTPRC, IRF4, INPP5D, PLCG2, HCLS1, ETS1] |
| Focal Adhesion | 11 | 5.88E-05 | 4.11 | [FGR, PDGFRA, FIGF, TNXB, RELN, TNN, TXK, COL4A4, PAK7, MET, THBS4] |
| Complement Activation, Classical Pathway | 6 | 8.38E-05 | 7.51 | [C3, C6, C7, C1S, C1R, MASP1] |

- 1. **Gene enrichment test using GOrilla on the gene list ranked by rank-sum test on LumA-R1 vs. LumA-R2**

To verify our results with a second tool for GO enrichment analysis, we first applied a rank-sum test on all dataset genes for testing difference in expression means between LumA-R1 and LumA-R2 samples. We then used the test pValues to rank the genes and applied the GOrilla[5] algorithm on the list composed of 19914 genes.

| GO Term | Description | Enrichment | FDR q-value |
| --- | --- | --- | --- |
| GO:0002376 | immune system process | 2.18 | 3.44E-49 |
| GO:0002682 | regulation of immune system process | 2.32 | 4.07E-47 |
| GO:0022610 | biological adhesion | 2.47 | 1.99E-40 |
| GO:0007155 | cell adhesion | 2.47 | 2.18E-40 |
| GO:0051239 | regulation of multicellular organismal process | 1.86 | 1.60E-38 |
| GO:0030155 | regulation of cell adhesion | 2.92 | 8.32E-38 |
| GO:0050865 | regulation of cell activation | 3.25 | 1.01E-37 |
| GO:0048583 | regulation of response to stimulus | 1.64 | 1.43E-37 |
| GO:0002684 | positive regulation of immune system process | 2.54 | 8.17E-36 |
| GO:0042127 | regulation of cell proliferation | 2.09 | 4.15E-34 |
| GO:0006955 | immune response | 2.25 | 4.52E-34 |
| GO:0048518 | positive regulation of biological process | 1.46 | 1.94E-33 |
| GO:0002694 | regulation of leukocyte activation | 3.24 | 2.97E-33 |
| GO:0007166 | cell surface receptor signaling pathway | 1.85 | 4.08E-32 |
| GO:0007165 | signal transduction | 1.49 | 4.15E-32 |
| GO:0051240 | positive regulation of multicellular organismal process | 2.09 | 2.36E-31 |
| GO:0051249 | regulation of lymphocyte activation | 3.33 | 1.33E-30 |
| GO:0050867 | positive regulation of cell activation | 3.69 | 1.37E-29 |
| GO:0001775 | cell activation | 2.74 | 1.64E-29 |
| GO:0034110 | regulation of homotypic cell-cell adhesion | 3.55 | 6.63E-29 |
| GO:0048584 | positive regulation of response to stimulus | 1.82 | 1.20E-28 |
| GO:0045785 | positive regulation of cell adhesion | 3.25 | 2.00E-28 |
| GO:0002696 | positive regulation of leukocyte activation | 3.67 | 3.34E-28 |
| GO:0098609 | cell-cell adhesion | 2.65 | 8.75E-28 |
| GO:0022407 | regulation of cell-cell adhesion | 3.18 | 3.87E-27 |
| GO:1903037 | regulation of leukocyte cell-cell adhesion | 3.46 | 4.20E-26 |
| GO:0051251 | positive regulation of lymphocyte activation | 3.68 | 1.49E-25 |
| GO:0050776 | regulation of immune response | 2.31 | 1.91E-25 |
| GO:0050863 | regulation of T cell activation | 3.46 | 3.05E-25 |
| GO:0045321 | leukocyte activation | 3.01 | 2.00E-24 |
| GO:0016337 | single organismal cell-cell adhesion | 2.85 | 1.22E-23 |
| GO:0050793 | regulation of developmental process | 1.71 | 1.54E-23 |
| GO:0034112 | positive regulation of homotypic cell-cell adhesion | 3.91 | 2.40E-23 |
| GO:0051094 | positive regulation of developmental process | 2.03 | 3.50E-23 |
| GO:0030154 | cell differentiation | 1.8 | 3.40E-23 |
| GO:1903039 | positive regulation of leukocyte cell-cell adhesion | 3.89 | 3.38E-23 |
| GO:0050870 | positive regulation of T cell activation | 3.89 | 1.48E-22 |
| GO:0008284 | positive regulation of cell proliferation | 2.26 | 1.84E-22 |
| GO:0098602 | single organism cell adhesion | 2.85 | 1.89E-22 |
| GO:0006952 | defense response | 1.96 | 2.37E-22 |
| GO:0048522 | positive regulation of cellular process | 1.42 | 3.21E-22 |
| GO:0046649 | lymphocyte activation | 3.14 | 5.71E-22 |
| GO:0022409 | positive regulation of cell-cell adhesion | 3.54 | 1.41E-21 |
| GO:0050896 | response to stimulus | 1.41 | 1.72E-21 |
| GO:0016477 | cell migration | 2.27 | 2.52E-21 |
| GO:0040011 | locomotion | 2.14 | 3.39E-21 |
| GO:0010033 | response to organic substance | 1.75 | 7.99E-21 |
| GO:0032101 | regulation of response to external stimulus | 2.14 | 1.51E-20 |
| GO:2000026 | regulation of multicellular organismal development | 1.81 | 1.61E-20 |
| GO:0002250 | adaptive immune response | 3.71 | 2.99E-20 |

1. **DNA methylation data analysis on all tumor types**

**Obtaining the DNA-methylation dataset and initial preprocessing**

Obtaining the data: TCGA's DNA-Methylation breast cancer dataset was downloaded from UCSC's Cancer Browser website. Samples were measure using Illumina's Infinium HumanMethylation450 BeadChip arrays.

Sample filtering: Started with 872 samples. Removed 8 gender/male, 5 sample type/metastatic, 19 tumor_tissue_site/NA, 98 sample type/normal, 33 PAM50 call/NA, 30 PAM50 Normal. Remained with 679 samples.

**Distribution of PAM50 labels in preprocessed Meth450 dataset:**

Total after preprocessing: 679

| Basal | 124 |
| --- | --- |
| Her2 | 42 |
| LumA | 378 |
| LumB | 135 |

Probeset filtering: The Illumina Methylation 450K array contains two type of probe chemistries that may require special normalization. To avoid dealing with integrating the two probe types and in order to zoom in CpGs characterizing known genes, we used only Infinium I probes that are also associated with a Gene symbol, keeping 107,639 probes for all further analyses.

Row Normalization: Rows were standardized (centered and normalized) before clustering was applied on the columns (samples) of the methylation beta matrix.

Sample Clustering: Kmeans using Correlation as distance metric.

|  | **Meth450 Clusters** | **Total** | **1** | **2** | **3** | **4** |
| --- | --- | --- | --- | --- | --- | --- |
|  |  | n=679 | n=182 | n=160 | n=174 | n=163 |
| **Age (Median)** |  | 58 | 62 | 58 | 56 | 54 |
| **ER Status** | NA | 38 ( 6%) | 12 ( 7%) | 7 ( 4%) | 11 ( 6%) | 8 ( 5%) |
|  | Negative | 144 ( 21%) | 11 ( 6%) | 3 ( 2%) | 6 ( 3%) | 124 ( 76%) |
|  | Positive | 497 ( 73%) | 159 ( 87%) | 150 ( 94%) | 157 ( 90%) | 31 ( 19%) |
| **PR Status** | NA | 41 ( 6%) | 12 ( 7%) | 8 ( 5%) | 11 ( 6%) | 10 ( 6%) |
|  | Negative | 202 ( 30%) | 43 ( 24%) | 11 ( 7%) | 18 ( 10%) | 130 ( 80%) |
|  | Positive | 436 ( 64%) | 127 ( 70%) | 141 ( 88%) | 145 ( 83%) | 23 ( 14%) |
| **Her2 Status** | NA | 232 ( 34%) | 57 ( 31%) | 41 ( 26%) | 71 ( 41%) | 63 ( 39%) |
|  | Negative | 394 ( 58%) | 92 ( 51%) | 115 ( 72%) | 95 ( 55%) | 92 ( 56%) |
|  | Positive | 53 ( 8%) | 33 ( 18%) | 4 ( 3%) | 8 ( 5%) | 8 ( 5%) |
| **PAM50** | Basal | 124 ( 18%) | 0 ( 0%) | 0 ( 0%) | 0 ( 0%) | 124 ( 76%) |
|  | Her2 | 42 ( 6%) | 14 ( 8%) | 0 ( 0%) | 3 ( 2%) | 25 ( 15%) |
|  | LumA | 378 ( 56%) | 96 ( 53%) | 119 ( 74%) | 156 ( 90%) | 7 ( 4%) |
|  | LumB | 135 ( 20%) | 72 ( 40%) | 41 ( 26%) | 15 ( 9%) | 7 ( 4%) |
| **Pathologic stage** | NA | 3 ( 0%) | 1 ( 1%) | 1 ( 1%) | 0 ( 0%) | 1 ( 1%) |
|  | Stage I | 112 ( 16%) | 28 ( 15%) | 21 ( 13%) | 43 ( 25%) | 20 ( 12%) |
|  | Stage II | 382 ( 56%) | 89 ( 49%) | 93 ( 58%) | 89 ( 51%) | 111 ( 68%) |
|  | Stage III | 172 ( 25%) | 61 ( 34%) | 43 ( 27%) | 40 ( 23%) | 28 ( 17%) |
|  | Stage IV | 6 ( 1%) | 2 ( 1%) | 1 ( 1%) | 1 ( 1%) | 2 ( 1%) |
|  | Stage X | 4 ( 1%) | 1 ( 1%) | 1 ( 1%) | 1 ( 1%) | 1 ( 1%) |
| **Histological type** | Infil. Ductal Carcinoma | 461 ( 68%) | 123 ( 68%) | 106 ( 66%) | 96 ( 55%) | 136 ( 83%) |
|  | Infil. Lobular Carcinoma | 140 ( 21%) | 41 ( 23%) | 33 ( 21%) | 62 ( 36%) | 4 ( 2%) |
|  | Medullary Carcinoma | 5 ( 1%) | 0 ( 0%) | 0 ( 0%) | 1 ( 1%) | 4 ( 2%) |
|  | Metaplastic Carcinoma | 2 ( 0%) | 0 ( 0%) | 0 ( 0%) | 0 ( 0%) | 2 ( 1%) |
|  | Mixed Histology | 24 ( 4%) | 6 ( 3%) | 11 ( 7%) | 5 ( 3%) | 2 ( 1%) |
|  | Mucinous Carcinoma | 14 ( 2%) | 7 ( 4%) | 2 ( 1%) | 5 ( 3%) | 0 ( 0%) |
|  | NA | 33 ( 5%) | 5 ( 3%) | 8 ( 5%) | 5 ( 3%) | 15 ( 9%) |

**Table S-6A:** Cohort description for the Methylation dataset analysis

**Survival and Recurrence KM plots for Meth450 samples based on PAM50 labels**

**Clustering Meth450 tumor samples to 4 using top 2000 variable CpGs (Inf I, GS only)**

|  | 5-year | Overall |
| --- | --- | --- |
| SURVIVAL | ****** |  |
| RECURRENCE |  |  |

1. **Methylation Luminal samples analysis**

**Clustering Meth450 Luminal tumor samples to 3 using top 2000 CpGs (Inf I, GS only)**

|  | **Meth450 Clusters** | **Total** | **1** | **2** | **3** |
| --- | --- | --- | --- | --- | --- |
|  |  | n=513 | n=127 | n=156 | n=230 |
| **Age (Median)** |  | 59 | 63 | 59 | 56 |
| **ER Status** | NA | 30 ( 6%) | 10 ( 8%) | 7 ( 4%) | 13 ( 6%) |
|  | Negative | 13 ( 3%) | 6 ( 5%) | 2 ( 1%) | 5 ( 2%) |
|  | Positive | 470 ( 92%) | 111 ( 87%) | 147 ( 94%) | 212 ( 92%) |
| **PR Status** | NA | 31 ( 6%) | 10 ( 8%) | 8 ( 5%) | 13 ( 6%) |
|  | Negative | 62 ( 12%) | 25 ( 20%) | 14 ( 9%) | 23 ( 10%) |
|  | Positive | 420 ( 82%) | 92 ( 72%) | 134 ( 86%) | 194 ( 84%) |
| **Her2 Status** | NA | 171 ( 33%) | 41 ( 32%) | 40 ( 26%) | 90 ( 39%) |
|  | Negative | 309 ( 60%) | 71 ( 56%) | 104 ( 67%) | 134 ( 58%) |
|  | Positive | 33 ( 6%) | 15 ( 12%) | 12 ( 8%) | 6 ( 3%) |
| **PAM50** | LumA | 378 ( 74%) | 76 ( 60%) | 98 ( 63%) | 204 ( 89%) |
|  | LumB | 135 ( 26%) | 51 ( 40%) | 58 ( 37%) | 26 ( 11%) |
| **Pathologic stage** | NA | 1 ( 0%) | 0 ( 0%) | 1 ( 1%) | 0 ( 0%) |
|  | Stage I | 92 ( 18%) | 22 ( 17%) | 18 ( 12%) | 52 ( 23%) |
|  | Stage II | 270 ( 53%) | 63 ( 50%) | 90 ( 58%) | 117 ( 51%) |
|  | Stage III | 143 ( 28%) | 39 ( 31%) | 46 ( 29%) | 58 ( 25%) |
|  | Stage IV | 4 ( 1%) | 2 ( 2%) | 1 ( 1%) | 1 ( 0%) |
|  | Stage X | 3 ( 1%) | 1 ( 1%) | 0 ( 0%) | 2 ( 1%) |
| **Histological type** | Infil. Ductal Carcinoma | 323 ( 63%) | 81 ( 64%) | 112 ( 72%) | 130 ( 57%) |
|  | Infil. Lobular Carcinoma | 136 ( 27%) | 34 ( 27%) | 26 ( 17%) | 76 ( 33%) |
|  | Medullary Carcinoma | 1 ( 0%) | 0 ( 0%) | 0 ( 0%) | 1 ( 0%) |
|  | Mixed Histology | 22 ( 4%) | 6 ( 5%) | 7 ( 4%) | 9 ( 4%) |
|  | Mucinous Carcinoma | 14 ( 3%) | 4 ( 3%) | 4 ( 3%) | 6 ( 3%) |
|  | NA | 17 ( 3%) | 2 ( 2%) | 7 ( 4%) | 8 ( 3%) |

**Table S-7A:** Cohort description for the Luminal Methylation dataset analysis

|  | Meth450 clusters | PAM50's LuminalA-LuminalB |
| --- | --- | --- |
| 5-Year SURVIVAL | ****** |  |
| 5-Year RECURRENCE |  |  |

|  | Meth450 clusters | PAM50's LuminalA-LuminalB |
| --- | --- | --- |
| OVERALL SURVIVAL | ****** |  |
| OVERALL RECURRENCE |  |  |

1. **Methylation Luminal-A samples analysis**

**Clustering Meth450 378 Luminal-A tumor samples to 3 using top 2000 CpGs , Inf 1, GS included only**

**Figure S-8A: Clustering the Luminal-A samples into 3 groups using DNA-Methylation data**

|  | **Meth450 Clusters** | **Total** | **1** | **2** | **3** |
| --- | --- | --- | --- | --- | --- |
|  |  | n=378 | n=84 | n=123 | n=171 |
| **Age (Median)** |  | 59 | 62 | 60 | 56 |
| **ER Status** | NA | 18 ( 5%) | 5 ( 6%) | 4 ( 3%) | 9 ( 5%) |
|  | Negative | 10 ( 3%) | 3 ( 4%) | 3 ( 2%) | 4 ( 2%) |
|  | Positive | 350 ( 93%) | 76 ( 90%) | 116 ( 94%) | 158 ( 92%) |
| **PR Status** | NA | 19 ( 5%) | 5 ( 6%) | 5 ( 4%) | 9 ( 5%) |
|  | Negative | 41 ( 11%) | 18 ( 21%) | 10 ( 8%) | 13 ( 8%) |
|  | Positive | 318 ( 84%) | 61 ( 73%) | 108 ( 88%) | 149 ( 87%) |
| **Her2 Status** | NA | 143 ( 38%) | 27 ( 32%) | 47 ( 38%) | 69 ( 40%) |
|  | Negative | 221 ( 58%) | 50 ( 60%) | 72 ( 59%) | 99 ( 58%) |
|  | Positive | 14 ( 4%) | 7 ( 8%) | 4 ( 3%) | 3 ( 2%) |
| **PAM50** | LumA | 378 (100%) | 84 (100%) | 123 (100%) | 171 (100%) |
| **Pathologic stage** | NA | 1 ( 0%) | 0 ( 0%) | 1 ( 1%) | 0 ( 0%) |
|  | Stage I | 77 ( 20%) | 14 ( 17%) | 18 ( 15%) | 45 ( 26%) |
|  | Stage II | 193 ( 51%) | 43 ( 51%) | 66 ( 54%) | 84 ( 49%) |
|  | Stage III | 103 ( 27%) | 27 ( 32%) | 37 ( 30%) | 39 ( 23%) |
|  | Stage IV | 2 ( 1%) | 0 ( 0%) | 1 ( 1%) | 1 ( 1%) |
|  | Stage X | 2 ( 1%) | 0 ( 0%) | 0 ( 0%) | 2 ( 1%) |
| **Histological type** | Infil. Ductal Carcinoma | 212 ( 56%) | 42 ( 50%) | 77 ( 63%) | 93 ( 54%) |
|  | Infil. Lobular Carcinoma | 127 ( 34%) | 32 ( 38%) | 36 ( 29%) | 59 ( 35%) |
|  | Mixed Histology | 17 ( 4%) | 4 ( 5%) | 5 ( 4%) | 8 ( 5%) |
|  | Mucinous Carcinoma | 9 ( 2%) | 3 ( 4%) | 1 ( 1%) | 5 ( 3%) |
|  | NA | 13 ( 3%) | 3 ( 4%) | 4 ( 3%) | 6 ( 4%) |

**Table S-8A:** Cohort description for the Luminal-A Methylation dataset analysis

**Figure S-8B:**  Comparison of the RNA-Seq based partition into LumA-R1/R2 and the Methylation based partition into LumA-M1/2/3

|  | **Survival** | **Recurrence** |
| --- | --- | --- |
| **Five-Year** | **** | **** |
| **Overall** | **** | **** |

**Figure S-8C: Survival analysis for the DNA methylation based partition of the Luminal-A samples**

1. **Differentially Methylated Gene Analysis (LumA-M1 vs. LumA-M2)**

We have generated a list of the top 1000 differentially methylated CpGs between LumA-M1 and LumA-M3 groups using rank-sum test having minimal median difference of 0.2.

The list represented 483 unique gene symbols for which gene enrichments were calculated using a background of 15737 genes included in the rank sum test.

- 1. **Gene Enrichment tests on the top 1000 differentially methylated CpGs**

The following results were obtained by using the Expander suite on a set of 429 (unique gene symbols having entrez Id) genes included in the set of 1000 differentially methylated CpGs:

**Gene Ontology enrichments detected using Expander TANGO on the list of 1000 DMGs**

| **Gene Ontology Term – Biological process** | **#Genes** | **Enrichment significance (pValue)** | **TANGO corrected pvalue** |
| --- | --- | --- | --- |
| system development - GO:0048731 | 188 | 9.86E-34 | 1.00E-05 |
| nervous system development - GO:0007399 | 132 | 4.38E-31 | 1.00E-05 |
| system process - GO:0003008 | 115 | 3.11E-27 | 1.00E-05 |
| neurological system process - GO:0050877 | 98 | 2.77E-26 | 1.00E-05 |
| multicellular organismal signaling - GO:0035637 | 72 | 8.17E-24 | 1.00E-05 |
| cell differentiation - GO:0030154 | 141 | 8.51E-23 | 1.00E-05 |
| pattern specification process - GO:0007389 | 52 | 4.10E-21 | 1.00E-05 |
| regionalization - GO:0003002 | 44 | 7.03E-21 | 1.00E-05 |
| brain development - GO:0007420 | 56 | 3.47E-20 | 1.00E-05 |
| neuron differentiation - GO:0030182 | 73 | 1.68E-19 | 1.00E-05 |
| regulation of multicellular organismal process - GO:0051239 | 104 | 6.24E-18 | 1.00E-05 |
| regulation of transcription from RNA polymerase II promoter - GO:0006357 | 85 | 2.44E-17 | 1.00E-05 |
| regulation of transcription, DNA-dependent - GO:0006355 | 151 | 6.31E-17 | 1.00E-05 |
| behavior - GO:0007610 | 45 | 9.98E-17 | 1.00E-05 |
| anatomical structure morphogenesis - GO:0009653 | 105 | 5.02E-16 | 1.00E-05 |
| central nervous system neuron differentiation - GO:0021953 | 26 | 5.79E-16 | 1.00E-05 |
| positive regulation of macromolecule biosynthetic process - GO:0010557 | 77 | 1.29E-15 | 1.00E-05 |
| organ morphogenesis - GO:0009887 | 61 | 1.81E-15 | 1.00E-05 |
| forebrain development - GO:0030900 | 36 | 2.34E-15 | 1.00E-05 |
| neuron fate commitment - GO:0048663 | 18 | 9.17E-15 | 1.00E-05 |
| positive regulation of nitrogen compound metabolic process - GO:0051173 | 77 | 2.91E-14 | 1.00E-05 |
| regulation of nervous system development - GO:0051960 | 43 | 3.61E-14 | 1.00E-05 |
| regulation of multicellular organismal development - GO:2000026 | 70 | 7.79E-14 | 1.00E-05 |
| cell differentiation in spinal cord - GO:0021515 | 15 | 2.65E-13 | 1.00E-05 |
| endocrine system development - GO:0035270 | 23 | 3.71E-13 | 1.00E-05 |
| ventral spinal cord development - GO:0021517 | 13 | 5.60E-13 | 1.00E-05 |
| regulation of cell differentiation - GO:0045595 | 64 | 1.35E-12 | 1.00E-05 |
| positive regulation of cellular process - GO:0048522 | 140 | 1.49E-12 | 1.00E-05 |
| embryo development - GO:0009790 | 59 | 1.80E-11 | 1.00E-05 |
| pancreas development - GO:0031016 | 16 | 5.74E-11 | 1.00E-05 |
| locomotion - GO:0040011 | 59 | 3.43E-10 | 1.00E-05 |
| limbic system development - GO:0021761 | 15 | 3.76E-10 | 1.00E-05 |
| negative regulation of biological process - GO:0048519 | 126 | 4.92E-10 | 1.00E-05 |
| pallium development - GO:0021543 | 17 | 8.01E-10 | 1.00E-05 |
| epithelium development - GO:0060429 | 39 | 1.83E-09 | 2.00E-05 |
| somitogenesis - GO:0001756 | 13 | 2.03E-09 | 2.00E-05 |
| hindbrain development - GO:0030902 | 17 | 3.97E-09 | 2.00E-05 |
| response to external stimulus - GO:0009605 | 60 | 9.70E-09 | 2.00E-05 |
| response to alkaloid - GO:0043279 | 15 | 1.96E-08 | 2.00E-05 |
| regulation of system process - GO:0044057 | 34 | 2.19E-08 | 2.00E-05 |
| negative regulation of developmental process - GO:0051093 | 35 | 4.35E-08 | 3.00E-05 |
| embryonic organ development - GO:0048568 | 28 | 4.52E-08 | 3.00E-05 |
| G-protein coupled receptor signaling pathway - GO:0007186 | 41 | 5.77E-08 | 5.00E-05 |
| regulation of neurological system process - GO:0031644 | 22 | 8.41E-08 | 8.00E-05 |

| **Gene Ontology Term – Molecular Function** | **#Genes** | **Enrichment significance (pValue)** | **TANGO corrected pvalue** |
| --- | --- | --- | --- |
| DNA binding - GO:0003677 | 125 | 1.06E-16 | 0.001 |
| regulatory region DNA binding - GO:0000975 | 38 | 6.08E-14 | 0.001 |
| neuron projection - GO:0043005 | 48 | 2.27E-11 | 0.001 |
| axon part - GO:0033267 | 19 | 2.73E-09 | 0.001 |

**KEGG PATHWAYS**

| **KEGG Pathway** | **#Genes** | **p-value** | **Enrichment** | **Genes** |
| --- | --- | --- | --- | --- |
| Neuroactive ligand-receptor interaction | 27 | 2.65E-10 | 4.2 | [CHRM2, VIPR2, GPR83, GRIK2, GRM1, CRHR2, GRIN2A, EDNRB, GRM7, GRM6, GALR1, NPBWR1, P2RY1, LEPR, NTSR1, PTGDR, DRD5, GHSR, GABBR2, GABRA5, GABRA4, HTR1A, SCTR, NMBR, SSTR4, GRIN1, GRIN3A] |
| Maturity onset diabetes of the young | 8 | 7.95E-08 | 12.8 | [NEUROD1, NR5A2, ONECUT1, SLC2A2, PAX6, NEUROG3, NKX2-2, FOXA2] |
| Calcium signaling pathway | 17 | 1.04E-05 | 3.41 | [RYR1, CHRM2, RYR2, PDE1C, PRKCB, CACNA1A, CACNA1E, RYR3, GRM1, GRIN1, GRIN2A, EDNRB, GNAL, CD38, NOS1, NTSR1, DRD5] |

- 1. **Gene enrichment test using Gorilla results for the top 1000 CpGs + 0.2 FC**

| GO Term | Description | FDR q-value | Enrichment |
| --- | --- | --- | --- |
| GO:0048856 | anatomical structure development | 6.07E-28 | 2.39 |
| GO:0032502 | developmental process | 1.98E-25 | 1.9 |
| GO:0032501 | multicellular organismal process | 9.55E-24 | 2.17 |
| GO:0044707 | single-multicellular organism process | 1.55E-22 | 2.15 |
| GO:0044700 | single organism signaling | 1.70E-21 | 3.72 |
| GO:0023052 | signaling | 1.89E-21 | 3.71 |
| GO:0007267 | cell-cell signaling | 1.70E-21 | 3.79 |
| GO:0030182 | neuron differentiation | 1.19E-20 | 6.57 |
| GO:0044767 | single-organism developmental process | 1.43E-19 | 1.84 |
| GO:0006357 | regulation of transcription from RNA polymerase II promoter | 1.21E-16 | 2.38 |
| GO:0007610 | behavior | 3.46E-16 | 3.95 |
| GO:0007389 | pattern specification process | 6.11E-16 | 4.49 |
| GO:0048869 | cellular developmental process | 1.89E-15 | 2.09 |
| GO:0021953 | central nervous system neuron differentiation | 1.93E-15 | 9.71 |
| GO:0003008 | system process | 4.97E-15 | 2.72 |
| GO:0007154 | cell communication | 5.80E-15 | 2.8 |
| GO:0050877 | neurological system process | 6.10E-15 | 3.23 |
| GO:0048731 | system development | 6.51E-15 | 3.39 |
| GO:0003002 | regionalization | 6.79E-15 | 5.42 |
| GO:0051239 | regulation of multicellular organismal process | 8.45E-15 | 2.1 |
| GO:0007268 | synaptic transmission | 1.07E-14 | 3.86 |
| GO:0045944 | positive regulation of transcription from RNA polymerase II promoter | 2.55E-14 | 2.78 |
| GO:0051960 | regulation of nervous system development | 4.17E-14 | 3.25 |
| GO:0030154 | cell differentiation | 7.25E-14 | 2.3 |
| GO:2000026 | regulation of multicellular organismal development | 7.99E-14 | 2.38 |
| GO:0006355 | regulation of transcription, DNA-templated | 1.55E-13 | 1.79 |
| GO:0044708 | single-organism behavior | 2.74E-13 | 4.1 |
| GO:1903506 | regulation of nucleic acid-templated transcription | 2.67E-13 | 1.78 |
| GO:0045893 | positive regulation of transcription, DNA-templated | 2.67E-13 | 2.39 |
| GO:1903508 | positive regulation of nucleic acid-templated transcription | 2.58E-13 | 2.39 |
| GO:0060284 | regulation of cell development | 3.36E-13 | 3.02 |
| GO:2001141 | regulation of RNA biosynthetic process | 3.79E-13 | 1.77 |
| GO:0051252 | regulation of RNA metabolic process | 4.36E-13 | 1.76 |
| GO:1902680 | positive regulation of RNA biosynthetic process | 7.11E-13 | 2.35 |
| GO:0031328 | positive regulation of cellular biosynthetic process | 9.91E-13 | 2.21 |

- 1. **Gene enrichments on the various subsets of differentially methylated CpGs between LumA-M1 and LumA-M3 subgroups**

|  | (1)  Hyper Meth. CpGs | | (2)  Neg: R < -0.2 | | (3)  Pos: R > 0.2 | |
| --- | --- | --- | --- | --- | --- | --- |
| Gene ontology | anatomical structure development | 6.1E-28 | developmental process | 7.8E-06 | pattern specification process | 1.1E-13 |
|  | developmental process | 2.0E-25 | single organism signaling | 2.4E-05 | regionalization | 1.1E-12 |
|  | multicellular organismal process | 9.6E-24 | signaling | 1.8E-05 | anatomical structure development | 2.2E-11 |
|  | single-multicellular organism process | 1.6E-22 | cellular developmental process | 1.4E-05 | single-organism developmental process | 1.9E-11 |
|  | single organism signaling | 1.7E-21 | single-organism developmental process | 2.3E-05 | anatomical structure morphogenesis | 1.8E-11 |
|  | Signaling | 1.9E-21 | anatomical structure development | 8.0E-05 | developmental process | 1.7E-11 |
|  | cell-cell signaling | 1.7E-21 | cell-cell signaling | 1.8E-04 | embryonic morphogenesis | 1.1E-10 |
|  | neuron differentiation | 1.2E-20 | cell differentiation | 2.2E-04 | cellular developmental process | 1.8E-10 |
|  | single-organism developmental process | 1.4E-19 | synaptic transmission | 4.4E-04 | organ development | 5.3E-10 |
|  | regulation of transcription from RNA polymerase II promoter | 1.2E-16 | anatomical structure morphogenesis | 6.1E-04 | single-multicellular organism process | 5.6E-10 |
|  | Behavior | 3.5E-16 | tube development | 1.8E-03 | cell fate commitment | 5.5E-10 |
|  | pattern specification process | 6.1E-16 | regulation of multicellular organismal development | 1.8E-03 | multicellular organismal process | 7.7E-10 |
|  | cellular developmental process | 1.9E-15 | cell development | 1.7E-03 | organ morphogenesis | 1.8E-09 |
|  | central nervous system neuron differentiation | 1.9E-15 | neuron differentiation | 2.0E-03 | transcription, DNA-templated | 6.5E-09 |
|  | system process | 5.0E-15 | regulation of nervous system development | 1.9E-03 | nucleic acid-templated transcription | 6.2E-09 |
| **Tumor Suppressor Gene (TSGene 2.0)** |  | 1.5E-03 |  | 9.7E-02 |  | 5.5E-02 |

**Table S-9.3: Gene enrichments on the various subsets of differentially methylated CpGs between LumA-M1 and LumA-M3 subgroups**.

- 1. **Feature enrichments on the various subsets of differentially methylated CpGs between LumA-M1 and LumA-M3 subgroups**

| **Group** |  | **Total** | **(1)**  **Hyper Meth. CpGs** | | **(2)**  **Neg: R < -0.2** | | **(3)**  **Pos: R > 0.2** | |
| --- | --- | --- | --- | --- | --- | --- | --- | --- |
| #CpGs |  | 94880 | 1000 | | 589 | | 212 | |
| **Label** | **Term** | **#Terms** | **#Terms** | **pValue** | **#Terms** | **pValue** | **#Terms** | **pValue** |
| **UCSC RefGene Group** | 1stExon | 9548 | 141 | 1.5E-04 | 104 | 1.4E-07 | 11 | 1.0E+00 |
|  | 3'UTR | 2489 | 11 | 1.0E+00 | 3 | 1.0E+00 | 13 | 1.8E-02 |
|  | 5'UTR | 11737 | 121 | 1.0E+00 | 82 | 3.2E-01 | 14 | 1.0E+00 |
|  | Body | 32979 | 285 | 1.0E+00 | 111 | 1.0E+00 | 141 | 9.5E-20 |
|  | TSS | 38127 | 442 | 1.6E-02 | 289 | 4.5E-05 | 33 | 1.0E+00 |
| **Regulatory Feature Group** | Gene Associated | 227 | 0 | 1.0E+00 | 0 | 1.0E+00 | 0 | 1.0E+00 |
|  | Gene Associated Cell type specific | 384 | 0 | 1.0E+00 | 0 | 1.0E+00 | 3 | 1.6E-01 |
|  | NonGene Associated | 472 | 2 | 1.0E+00 | 0 | 1.0E+00 | 0 | 1.0E+00 |
|  | NonGene Associated Cell type specific | 40 | 4 | 2.8E-03 | 1 | 4.9E-01 | 1 | 2.2E-01 |
|  | Promoter Associated | 36454 | 41 | 1.0E+00 | 95 | 1.0E+00 | 6 | 1.0E+00 |
|  | Promoter Associated Cell type specific | 1676 | 9 | 1.0E+00 | 26 | 1.4E-04 | 0 | 1.0E+00 |
|  | Unclassified | 7559 | 71 | 1.0E+00 | 73 | 5.8E-04 | 17 | 1.0E+00 |
|  | Unclassified Cell type specific | 7962 | 211 | 8.8E-35 | 86 | 3.9E-06 | 50 | 1.3E-10 |
|  | Unassigned | 40106 | 662 | 7.4E-52 | 308 | 4.9E-06 | 135 | 1.8E-09 |
| **DMR** (Differentially Methylated Region) | CDMR (Cancer DMR) | 855 | 44 | 1.5E-16 | 14 | 3.9E-03 | 20 | 1.1E-13 |
|  | DMR | 6722 | 391 | 9.2E-183 | 195 | 1.7E-75 | 54 | 1.4E-15 |
|  | RDMR (Reprogramming DMR) | 1447 | 33 | 1.9E-04 | 14 | 1.8E-01 | 22 | 2.2E-11 |
|  | Unassigned | 85856 | 532 | 1.0E+00 | 366 | 1.0E+00 | 116 | 1.0E+00 |
| **Enhancer** |  | 10107 | 99 | 1.2E-09 | 66 | 8.0E-06 | 29 | 1.7E-04 |
| **DHS** |  | 17152 | 137 | 1.1E-07 | 86 | 2.1E-03 | 44 | 1.7E-05 |
| **Tumor Suppressor Gene (TSGene 2.0)** |  | 944 | 48 | 1.5E-03 | 29 | 9.7E-02 | 14 | 5.5E-02 |

**Table S-9.4: Feature enrichments on the various subsets of differentially methylated CpGs between LumA-M1 and LumA-M3 subgroups**. Group 1 is composed of the 1000 top differentially methylated CpGs exhibiting mean difference of at least 0.2. All the CpGs on this list showed significant hyper-methylation on the LumA-M1 samples compared to LumA-M3 samples. Group 2 is composed of the 589 CpGs exhibting differential methylation pValue<0.01, methylation mean difference>0.2 and spearman based correlation to expression that is lower than 0.2. Group 3 212 CpGs exhibiting differential methylation pValue<0.01, methylation mean difference>0.2 and spearman based correlation to expression that is higher than 0.2. All p-values represent hyper-geometric based over-representation and are FDR corrected.

|  |  | Hyper Meth. CpGs | | Neg: R < -0.2 | | Pos: R > 0.2 | |
| --- | --- | --- | --- | --- | --- | --- | --- |
| **Label** | **Term** | **Over-representation FDR corrected pValue** | **Under-representation FDR corrected pValue** | **Over-representation FDR corrected pValue** | **Under-representation FDR corrected pValue** | **Over-representation FDR corrected pValue** | **Under-representation FDR corrected pValue** |
| **UCSC RefGene Group** | 1stExon | **1.E-04** | 1.E+00 | **1.E-07** | 1.E+00 | 1.E+00 | 3.E-02 |
|  | 3'UTR | 1.E+00 | 2.E-03 | 1.E+00 | **6.E-04** | 2.E-02 | 1.E+00 |
|  | 5'UTR | 1.E+00 | 8.E-01 | 3.E-01 | 1.E+00 | 1.E+00 | 2.E-02 |
|  | Body | 1.E+00 | **7.E-05** | 1.E+00 | **1.E-16** | **9.E-20** | 1.E+00 |
|  | TSS | 2.E-02 | 1.E+00 | **4.E-05** | 1.E+00 | 1.E+00 | **7.E-14** |
| **Regulatory Feature Group** | Gene Associated | 1.E+00 | 2.E-01 | 1.E+00 | 5.E-01 | 1.E+00 | 1.E+00 |
|  | Gene Associated Cell type specific | 1.E+00 | 5.E-02 | 1.E+00 | 2.E-01 | 2.E-01 | 1.E+00 |
|  | NonGene Associated | 1.E+00 | 3.E-01 | 1.E+00 | 1.E-01 | 1.E+00 | 8.E-01 |
|  | NonGene Associated Cell type specific | 3.E-03 | 1.E+00 | 5.E-01 | 1.E+00 | 2.E-01 | 1.E+00 |
|  | Promoter Associated | 1.E+00 | **2.E-146** | 1.E+00 | **3.E-31** | 1.E+00 | **4.E-34** |
|  | Promoter Associated Cell type specific | 1.E+00 | 5.E-02 | **1.E-04** | 1.E+00 | 1.E+00 | 7.E-02 |
|  | Unclassified | 1.E+00 | 4.E-01 | 6.E-04 | 1.E+00 | 1.E+00 | 1.E+00 |
|  | Unclassified Cell type specific | 9.E-35 | 1.E+00 | 4.E-06 | 1.E+00 | 1.E-10 | 1.E+00 |
|  | Unassigned | 7.E-52 | 1.E+00 | 5.E-06 | 1.E+00 | 2.E-09 | 1.E+00 |
| **Relation to UCSC CpG Island** | Island | 1.E+00 | 9.E-04 | 1.E+00 | 1.E-03 | 1.E+00 | 7.E-02 |
|  | N_Shelf | 1.E+00 | 5.E-01 | 1.E+00 | 7.E-01 | 1.E+00 | 1.E+00 |
|  | N_Shore | 6.E-02 | 1.E+00 | 4.E-02 | 1.E+00 | 7.E-01 | 1.E+00 |
|  | S_Shelf | 8.E-02 | 1.E+00 | 8.E-02 | 1.E+00 | 1.E+00 | 9.E-01 |
|  | S_Shore | 3.E-02 | 1.E+00 | 3.E-02 | 1.E+00 | 4.E-01 | 1.E+00 |
|  | Unassigned | 4.E-01 | 1.E+00 | 7.E-01 | 1.E+00 | 2.E-01 | 1.E+00 |
| **DMR** (Differentially Methylated Region | CDMR | **2.E-16** | 1.E+00 | 4.E-03 | 1.E+00 | 1.E-13 | 1.E+00 |
|  | DMR | **9.E-183** | 1.E+00 | 2.E-75 | 1.E+00 | 1.E-15 | 1.E+00 |
|  | RDMR | **2.E-04** | 1.E+00 | 2.E-01 | 1.E+00 | 2.E-11 | 1.E+00 |
|  | Unassigned | 1.E+00 | **2.E-205** | 1.E+00 | 2.E-75 | 1.E+00 | 5.E-40 |
| **Enhancer** | 0 | 1.E+00 | 1.E-09 | 1.E+00 | 8.E-06 | 1.E+00 | 2.E-04 |
|  | 1 | **1.E-09** | 1.E+00 | 8.E-06 | 1.E+00 | 2.E-04 | 1.E+00 |
| **DHS** | 0 | 1.E+00 | 1.E-07 | 1.E+00 | 2.E-03 | 1.E+00 | 2.E-05 |
|  | 1 | **1.E-07** | 1.E+00 | 2.E-03 | 1.E+00 | 2.E-05 | 1.E+00 |
| **Tumor Suppressor Gene Catalogue (TSG 2.0)** | 0 | 1.E+00 | 2.E-03 | 1.E+00 | 1.E-01 | 1.E+00 | 6.E-02 |
|  | 1 | **2.E-03** | 1.E+00 | 1.E-01 | 1.E+00 | 6.E-02 | 1.E+00 |

**Table S-9.5: Feature enrichments on the various subsets of differentially methylated CpGs between LumA-M1 and LumA-M3 subgroups** (Including under-representation pValues)

1. Cox proportional hazards model analysis

|  | **Survival** | | | | **Recurrence** | | | |
| --- | --- | --- | --- | --- | --- | --- | --- | --- |
|  | **Univariate** | | ***Multivariate*** | | **Univariate** | | ***Multivariate*** | |
| **Variable** | ***HR*** | ***pValue*** | ***HR*** | ***pValue*** | ***HR*** | **pValue** | ***HR*** | ***pValue*** |
| ***LumA-R (1 vs 2)*** | 0.44 | 0.10939 | 0.56 | 0.36991 | **0.20** | **0.00421** | **0.06** | **0.00693** |
| ***LumA-M (2,3 vs 1)*** | **4.53** | **0.00258** | **6.68** | **0.00484** | 1.64 | 0.34338 | 3.04 | 0.07028 |
| *Age (<60 vs.>=60 years)* | **5.79** | **0.00624** | **11.20** | **0.0037** | 2.18 | 0.10301 | 1.03 | 0.96530 |
| *Pathologic stage (I,II vs. III,IV)* | 1.30 | 0.62799 | 2.12 | 0.25519 | 2.09 | 0.11941 | 1.93 | 0.26992 |
| *ER Status* | 1.72 | 0.60363 | 7.17 | 0.18095 | 0.00 | 0.99217 | 0.00 | 0.99575 |
| *PR Status* | 1.03 | 0.96671 | 0.47 | 0.50039 | 0.37 | 0.33789 | 0.29 | 0.29092 |
| *Her2 Status* | 0.79 | 0.8208 | 1.48 | 0.72659 | 0.99 | 0.98916 | 0.64 | 0.68789 |

**Table S-10A:** Univariate and Multivariate Cox analysis of Luminal-A subgroups for five-year survival and five-year recurrence.

1. Joint clustering of Luminal-A samples using both expression and DNA methylation datasets

After establishing that Luminal-A samples (as labeled by PAM50) can be further divided into distinct clinically meaningful subgroups by either the RNA-Seq or the methylation datasets separately, we set out to generate a single robust Luminal-A partition that would leverage from the complementary biological information stored in both expression and methylation datasets.

To this end, we unified both expression and methylation datasets into a single “bi-omic” dataset composed of 378 Luminal-A samples for which both types of data are available. From each dataset we selected the top 1000 variable features (top 1000 variable genes from the RNA-Seq dataset, and top 1000 variable CpGs from the methylation dataset). We then clustered the samples using a variant of K-Means for which the distance metric is formulated as the average of the correlation based distances on the two data types, i.e., for samples s, t

$D_{st}=\frac{d_{st}^{Exp}+d_{st}^{Meth}}{2}$ where the distance $d_{st}$ for each data type is 1 minus the correlation between the 1000-long vectors of samples s and t.

Interestingly, applying the method on the Luminal-A samples did not produce an improved partition neither in terms of stability (repeated executions yielded significantly different results) nor in terms of survival prediction compared with the separate partitions. We assume this result can be attributed to fact that the two datasets impose very different partitions on the samples, making this dataset sub-optimal target for the described integrative clustering approach.

**Figure S-11A:** Clustering of 378 Luminal-A samples to 3 using K-Means algorithm based on top 1000 variably expressed genes and top 1000 variably methylated CpG islands.

1. LumA-R1/2 clusters are enriched for the ILC classes defined by TCGA

We compared our RNA-Seq based partition of the Luminal-A samples to the three ILC (Invasive Lobular Carcinoma) classes recently defined by TCGA [7]. A Chi-square test determined that the two partitions are related (p=1.2e-04, based on the 104 ILC samples appearing on both datasets). The hyper-geometric test we used to evaluate enrichment of specific ILC classes within each of our Luminal clusters. LumA-R1 cluster was found to be significantly enriched for the proliferative ILC class (p=8.1e-04), whereas the LumA-R2 cluster was found to be significantly enriched for the Reactive-like ILC class (2.4-e04).

1. **LumA-M1 samples are enriched for the Epi-LumB group identified by Stefansson et al.**

For comparing our methylation based Luminal-A clusters to the bad outcome Luminal group described by Stefansson et al.[6] (named Epi-LumB as it was largely composed of Luminal-B samples), we first kept only samples that appeared both in our partition and in Epi-LumB labels for TCGA's Meth450 dataset, and then we calculated enrichment for the Epi-LumB label in our clusters.

Cluster LumA-M1 was found to be enriched with the Epi-LumB label (p=1.6e-07), enforcing our observation that this group is associated with a bad outcome (though labeled as Luminal-A by PAM50).

**References**

[1] I. Ulitsky, A. Maron-Katz, S. Shavit, D. Sagir, C. Linhart, R. Elkon, A. Tanay, R. Sharan, Y. Shiloh, and R. Shamir, “Expander: from expression microarrays to networks and functions.,” *Nat. Protoc.*, vol. 5, no. 2, pp. 303–22, Mar. 2010.

[2] M. Ashburner, C. A. Ball, J. A. Blake, D. Botstein, H. Butler, J. M. Cherry, A. P. Davis, K. Dolinski, S. S. Dwight, J. T. Eppig, M. A. Harris, D. P. Hill, L. Issel-Tarver, A. Kasarskis, S. Lewis, J. C. Matese, J. E. Richardson, M. Ringwald, G. M. Rubin, and G. Sherlock, “Gene ontology: tool for the unification of biology. The Gene Ontology Consortium.,” *Nat. Genet.*, vol. 25, no. 1, pp. 25–9, May 2000.

[3] M. Kanehisa and S. Goto, “KEGG: kyoto encyclopedia of genes and genomes.,” *Nucleic Acids Res.*, vol. 28, no. 1, pp. 27–30, Jan. 2000.

[4] T. Kelder, M. P. van Iersel, K. Hanspers, M. Kutmon, B. R. Conklin, C. T. Evelo, and A. R. Pico, “WikiPathways: building research communities on biological pathways.,” *Nucleic Acids Res.*, vol. 40, no. Database issue, pp. D1301–7, Jan. 2012.

[5] E. Eden, R. Navon, I. Steinfeld, D. Lipson, and Z. Yakhini, “GOrilla: a tool for discovery and visualization of enriched GO terms in ranked gene lists.,” *BMC Bioinformatics*, vol. 10, no. 1, p. 48, Jan. 2009.

[6] O. a. Stefansson, S. Moran, A. Gomez, S. Sayols, C. Arribas-Jorba, J. Sandoval, H. Hilmarsdottir, E. Olafsdottir, L. Tryggvadottir, J. G. Jonasson, J. Eyfjord, and M. Esteller, “A DNA methylation-based definition of biologically distinct breast cancer subtypes,” *Mol. Oncol.*, vol. 9, pp. 555–568, Nov. 2015.

[7] G. Ciriello, M. L. L. Gatza, A. H. H. Beck, M. D. D. Wilkerson, S. K. K. Rhie, A. Pastore, H. Zhang, M. McLellan, C. Yau, C. Kandoth, R. Bowlby, H. Shen, S. Hayat, R. Fieldhouse, S. C. C. Lester, G. M. K. M. K. Tse, R. E. E. Factor, L. C. C. Collins, K. H. H. Allison, Y.-Y. Chen, K. Jensen, N. B. B. Johnson, S. Oesterreich, G. B. B. Mills, A. D. D. Cherniack, G. Robertson, C. Benz, C. Sander, P. W. W. Laird, K. A. A. Hoadley, T. A. A. King, and C. M. Perou, “Comprehensive Molecular Portraits of Invasive Lobular Breast Cancer,” *Cell*, vol. 163, no. 2, pp. 506–519, Oct. 2015.
